# Isc10, an inhibitor of the Smk1 MAPK, prevents activation loop autophosphorylation and substrate phosphorylation through separate mechanisms

**DOI:** 10.1016/j.jbc.2022.102450

**Published:** 2022-09-03

**Authors:** Abhimannyu Rimal, Thomas M. Swayne, Zeal P. Kamdar, Madison A. Tewey, Edward Winter

**Affiliations:** Department of Biochemistry and Molecular Biology, Thomas Jefferson University, Philadelphia, Pennsylvania, USA

**Keywords:** mitogen-activated protein kinase, meiosis, activation-loop autophosphorylation, yeast, Smk1, Cak1/Ssp2, Isc10, BD, binding domain, IPA, inhibited/poised/active, KAD, kinase-activating domain, MII, meiosis II, MS, mass spectrometry, RRM, RNA recognition motif, TCA, trichloroacetic acid, WT, wild-type

## Abstract

Many eukaryotic protein kinases are activated by the intramolecular autophosphorylation of activation loop residues. Smk1 is a meiosis-specific mitogen-activated protein kinase (MAPK) in yeast that autophosphorylates its activation loop tyrosine and thereby upregulates catalytic output. This reaction is controlled by an inhibitor, Isc10, that binds the MAPK during meiosis I and an activator, Ssp2, that binds Smk1/Isc10 during meiosis II. Upon completion of the meiotic divisions, Isc10 is degraded, and Smk1 undergoes autophosphorylation to generate the high activity form of the MAPK that controls spore formation. How Isc10 inhibits Smk1 is not clear. Here, we use a bacterial coexpression/reconstitution system to define a domain in the carboxy-terminal half of Isc10 that specifically inhibits Smk1 autophosphorylation. Nevertheless, Smk1 bound by this domain is able to phosphorylate other substrates, and it phosphorylates the amino-terminal half of Isc10 on serine 97. In turn, the phosphorylated motif in Isc10 inhibits the Smk1 active site. These data show that Isc10 inhibits autophosphorylation and the phosphorylation of substrates by separate mechanisms. Furthermore, we demonstrate Isc10 can inhibit the autophosphorylation of the mammalian intestinal cell kinase ICK1 (also known as CILK1), suggesting a conserved mechanism of action. These findings define a novel class of developmentally regulated molecules that prevent the self-activation of MAPKs and MAPK-like enzymes.

Autophosphorylation of the activation loop is a common mechanism that upregulates eukaryotic protein kinases (1). In some cases, activation loops are phosphorylated by upstream kinases, while in other cases, they are autophosphorylated. This latter mechanism of kinase activation is widespread, influencing the activity of at least a third of the human kinome.

Activation loop autophosphorylation plays an especially prominent role in regulating CMGC group kinases (cyclin dependent kinases, mitogen-activated protein kinases, glycogen synthase kinases, and CDK-like kinases). In most cases, these reactions occur by an intramolecular (in-*cis*) mechanism. Although mature CMGC group kinases typically phosphorylate downstream substrates on serine/threonine (S/T) residues, the most commonly autophosphorylated activation loop residue is tyrosine (Y). It has been proposed that these reactions involve a transitional “Prone-to-Autophosphorylate” (PA) conformation (1). In several cases, autophosphorylation of activation loop Y-residues has been shown to take place cotranslationally or shortly after translation has taken place when the kinase is associated with the Hsp90/Cdc37 chaperone complex (2, 3, 4, 5). More recently, it has been shown that activation loop autophosphorylation of CMGC kinases requires hydroxylation of a proline located in the CMGC/MAPK-insert, a distinct segment located in the C-lobe of most CMGC kinases (6). It has also been shown that mutations that weaken the catalytic spine of the Erk2 MAPK can increase the *cis*-autophosphorylation of its activation loop Y (7, 8). Taken together, these findings suggest that the *cis*-autophosphorylation of activation loop Y-residues is an evolutionarily ancient reaction that is repressed in some CMGC kinases yet not in others and that this mechanism enhances the regulatory repertoire of this critical group of signaling enzymes.

The sporulation-specific MAPK, Smk1, is a meiosis-specific kinase in yeast that autophosphorylates its activation loop tyrosine (Y209) upon completion of the meiotic divisions (9). Once activated, Smk1 triggers key steps in spore assembly (10, 11). Previous studies have revealed that Smk1 is controlled by the CDK activating kinase (Cak1) (12, 13), an activator (Ssp2) (14, 15), and an inhibitor (Isc10) (16). In addition, the anaphase promoting complex/cyclosome (APC/C) E3 ubiquitin-ligase bound to a meiosis-specific activator, Ama1, triggers Smk1 activation as cells complete meiosis II (MII) (17, 18). These findings have led to a model in which Smk1 is first bound by the Isc10 inhibitor protein during MI to form “inhibited” (I) complexes. Cak1, which is produced as a constitutively active enzyme (19, 20, 21), phosphorylates Smk1 on its activation loop T at this stage (9). During MII, the monophosphorylated I complexes are bound by the activator, Ssp2, to form “poised” (P) complexes. As cells complete MII, the APC/C^Ama1^ is activated. This leads to the ubiquitylation and degradation of the Isc10 protein, releasing “active” (A) complexes (Smk1/Ssp2) that undergo autophosphorylation. The doubly phosphorylated Smk1 bound to Ssp2 then triggers key steps in spore morphogenesis. The “inhibited/poised/active (IPA)” model for Smk1 activation thereby provides an explanation for how key steps in spore differentiation take place only after MII has been completed (16).

This study was designed to investigate how Isc10 inhibits Smk1. We used a heterologous bacterial expression system, *in vitro* biochemical reactions, and *Saccharomyces cerevisiae* undergoing meiotic development to show that Isc10 inhibits Smk1 through two mechanisms. First, a C-terminal segment in Isc10 specifically inhibits autophosphorylation of Smk1’s activation loop on Y209. This segment is referred to as the Y Autophosphorylation Inhibitor motif, or YAI hereafter. Although the YAI reduces the catalytic activity of Smk1 by preventing activation loop autophosphorylation, Smk1 that has not undergone autophosphorylation has a modest level of catalytic activity and Smk1 bound to the YAI can phosphorylate substrates. We find that Smk1 bound to Isc10 and Ssp2 phosphorylates the Isc10 protein on serine 97. The phosphorylated S97 motif in turn prevents Smk1 from phosphorylating Isc10 on other residues. We also find that an Smk1-Y209F mutant is not inhibited by the pS97 motif, suggesting that the Y209 hydroxyl plays an essential role in pS97-mediated inhibition. These findings suggest that once Smk1 undergoes autophosphorylation, it can no longer be inhibited by Isc10, implying a switch-like role for Isc10 in triggering activation of the MAPK. Our findings indicate that this switch operates during yeast meiotic development since expression of the YAI during meiosis in yeast downregulates Smk1 autophosphorylation. Interestingly, we find that Isc10 also inhibits the autophosphorylation of the activation loop Y of the mammalian intestinal cell kinase (ICK1).

## Results

### A domain in the C-terminal half of Isc10 inhibits Smk1 autophosphorylation

**Figure 1:**
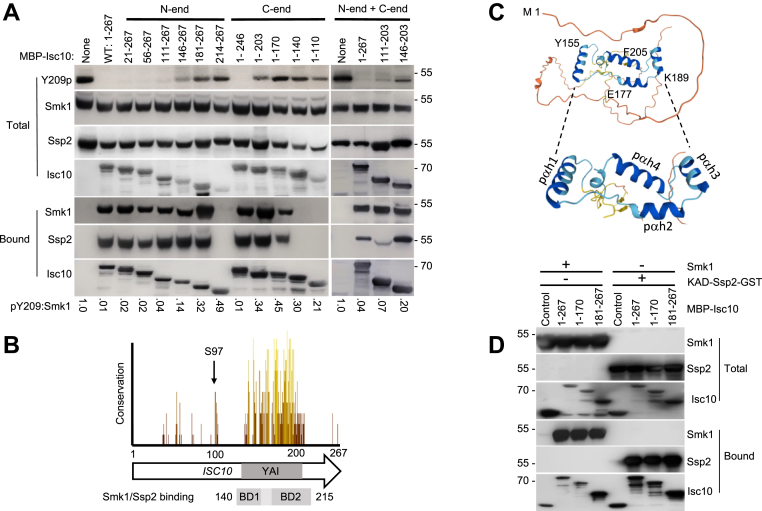
**An evolutionarily conserved segment of Isc10 inhibits Smk1 autophosphorylation.***A*, extracts from bacterial cells expressing Smk1, Ssp2^KAD^-GST, Cak1, and different forms of MBP-tagged Isc10 deleted from the N-end, the C-end, or from both ends as indicated were resolved by electrophoresis and analyzed by immunoblotting with antibodies directed against pY209, Smk1, GST (Ssp2), and MBP (Isc10) (Total). The residues of Isc10 remaining in each of the MBP-Isc10 constructs are indicated (MBP-Isc10, above). The ratios of the pY209 to Smk1 immunoreactivity signals were quantified from the upper two images for each sample and normalized to the ratio of the sample lacking Isc10 which was set at 1.0 (pY209:Smk1, below). A fraction of each sample was also bound to amylose-agarose beads to enrich for MBP-Isc10 and analyzed with the indicated antibodies (Bound). *B*, conservation of Isc10 amino acid sequences across *Saccharomycetes*. Bar heights and color (*brown*-low, *yellow*-high) indicate the extent of conservation. The YAI motif is shown as a *gray* rectangle with the bipartite binding domains (BDs) 1 and 2 indicated based on the abilities of Isc10 deletants to bind Smk1/Ssp2. *C*, prediction of Isc10 structure by AlphaFold with an enlarged view (dotted lines) of the region with the highest confidence structure (in *blue*). The numbers indicate the first amino acids in pαhs1-4. *D*, interaction of BD1 and BD2 with Smk1 and Ssp2. MBP fused to full-length Isc10 (1–267), an Isc10 construct lacking pαhs 2 to 4 (1–170) or an Isc10 construct lacking all of pαh 1 and part of pαh 2 (180–267) were coexpressed with Smk1 (left) or Ssp2 (right). The total soluble extract (Total) and material that was retained on amylose-agarose beads (Bound) are indicated. The values to the right and left of panels (*A*) and (*D*), respectively, show the positions of molecular weight standards in kDa.

Smk1 autophosphorylation can be studied using a heterologous bacterial system in which Smk1, Ssp2, and Cak1 are coexpressed in various combinations (14). In this system, Ssp2 is required for Smk1 catalytic activity and autophosphorylation of Smk1’s activation loop tyrosine (Y209). We showed that the kinase-activating domain (KAD) of Ssp2 is contained in the C-terminal half (residues 137–371) of Ssp2. In the experiments described here, we used Ssp2^137-371^ fused to glutathione-*S*-transferase (Ssp2^KAD^-GST). Cak1, which phosphorylates Smk1’s activation loop threonine (T207), modestly increases the Ssp2-dependent autophosphorylation of Smk1 on Y209. The expression of Isc10 eliminates almost all Y209 autophosphorylation in the presence or absence of Cak1 (16). The inhibition of Y209p autophosphorylation by Isc10 can be observed in Fig. 1*A* (compare the first two lanes in the top image). These findings reflect the roles of Ssp2, Cak1, and Isc10 in controlling the activation of Smk1 in meiotic yeast cells (16).

To define the region of Isc10 that inhibits Smk1 autophosphorylation, we assayed deletions in the MBP-Isc10 construct. Removal of residues 1 to 110 from the N terminus of Isc10 (Isc10^111-267^) had little effect on Isc10’s ability to inhibit Smk1 autophosphorylation (Fig. 1*A*, N-end deletions). Removal of an additional 35 residues (Isc10^146-267^) reduced the ability of Isc10 to inhibit Smk1 autophosphorylation (pY209 becomes detectable). Deletion of 21 residues from the C terminus of Isc10 (Isc10^1-246^) did not affect Isc10’s ability to inhibit Smk1 autophosphorylation, while deletion of an additional 44 residues (Isc10^1-203^) reduced Isc10’s inhibitory activity (Fig. 1*A*, C-end deletions). These data show that the Y209 Autophosphorylation Inhibitory (YAI) segment resides between amino acids 110 and 246 of Isc10. Notably, this segment of Isc10 contains a region with a relatively high degree of evolutionary conservation across the *Saccharomycetes* family while the rest of Isc10 is poorly conserved (Fig. 1*B*). Moreover, the AlphaFold program predicts that the conserved region of Isc10 contains a high confidence secondary structure while it does not predict significant secondary structural features in the rest of the protein (22, 23) (Fig. 1*C*). The major secondary structural features within this segment are four predicted α-helices (labeled pαhs 1–4 in Fig. 1*C*).

While pαh’s 1 to 3 are highly conserved across *Saccharomycetes* species, pαh4 is not as conserved. We wondered whether pαh4 is important for YAI activity only in the full-length Isc10 protein but not in an Isc10 protein lacking the nonconserved amino-terminal segment. To test this, we generated a deleted Isc10 protein lacking both pαh4 and the first 110 nonconserved residues. This segment of Isc10 (Isc10^111-203^) was almost as effective as the full-length Isc10 protein in inhibiting the autophosphorylation of Smk1 on Y209 (Fig. 1*A*, right, N-end + C-end deletants). Moreover, further deletion of residues 111 to 145 to generate a fragment of Isc10 that contains only pαh’s 1 to 3 (Isc10^146-203^) retained partial YAI activity. pαh4’s importance for YAI activity when the first 110 residues of Isc10 are present but not when they are absent might suggest that this segment of Isc10 can modify the activity of the YAI through pαh4. Irrespective, these findings show that pαh’s 1 to 3 in the Isc10^146-203^ are sufficient for core YAI functionality.

### The YAI binds Smk1/Ssp2 through a bipartite-binding domain

To assay the collection of MBP-Isc10 truncated mutants for binding to Ssp2 and Smk1, we purified the MBP-Isc10 proteins using amylose beads. Smk1 and Ssp2 that copurified with MBP-Isc10 in these samples were measured by immunoblotting (bound samples in Fig. 1*A*). All Isc10 fragments that inhibited Smk1 autophosphorylation formed complexes with Smk1 and Ssp2 that were stable to purification. However, some of the Isc10 mutants that did not fully inhibit Smk1 autophosphorylation also formed complexes with Smk1/Ssp2. Specifically, while the N-terminal truncated Isc10^181-267^ protein did not prevent autophosphorylation, it still formed complexes with Smk1/Ssp2. This suggests that while the most N-terminal helix predicted by AlphaFold (pαh1 [residues 155–168]) is required to inhibit Smk1 autophosphorylation, it is not essential for binding to Smk1/Ssp2 since pαh1 is not present in the binding competent but functionally defective deletant. Similarly, while Isc10’s inhibitory activity was significantly reduced when 64 amino acids were removed from the C terminus (the Isc10^1-203^ protein in Fig. 1*A*), this mutant and even a mutant lacking 97 C-terminal residues (Isc10^1-170^) still bound Smk1/Ssp2. These findings suggest that pαhs 2, 3, and 4, are not required for Isc10 to bind Smk1/Ssp2 since they are not present in the binding competent but functionally defective Isc10^1-170^ protein. Indeed, binding was not eliminated from the C-terminally deleted proteins until an additional 30 residues, which contains the entirety of pαh1, were deleted from the Isc10^1-170^ protein (generating Isc10^1-140^). Collectively, these findings suggest that Isc10 binds Smk1/Ssp2 through two separate binding domains (BDs 1 and 2 as shown in the bottom of Fig. 1*B*) and that both BDs are required to inhibit Smk1 autophosphorylation. The data indicate that BD1 is within pαh1 and that BD2 is within the pαh2/3/4 segment (Fig. 1, *B* and *C*). Because pαh4 is not required for YAI functionality, we speculate that BD2 is within pαh2/3.

### BDs 1 and 2 can each bind Smk1 and Ssp2 in the absence of the other protein

To determine whether BD1 and BD2 can individually bind Smk1 and Ssp2, MBP fused to Isc10 residues 1 to 170 that contains BD1 or to Isc10 residues 181 to 267 that contains BD2, were produced in bacteria that coexpressed Smk1 or Ssp2. The MBP-Isc10 deletants were purified from bacterial extracts, and the presence of Smk1 or Ssp2 were tested (Fig. 1*D*). These assays show that BD1 and BD2 can each form complexes with Smk1 or Ssp2 in the absence of the other protein. Whether these interactions reflect mutually exclusive (potentially competitive) or simultaneous interactions in the poised Isc10/Smk1/Ssp2 complex remain to be determined.

### The YAI motif inhibits Smk1 autophosphorylation in yeast

**Figure 2:**
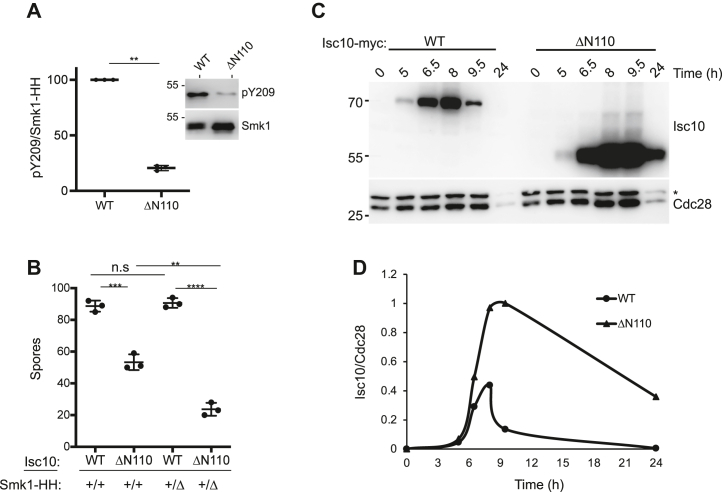
**The YAI inhibits Smk1 autophosphorylation in yeast.***A*, Isc10-Δ110 inhibits Smk1 autophosphorylation. Cells were collected 8 h after being transferred to sporulation medium when MII had been completed in >75% of cells as judged by DAPI staining. Smk1 tagged with an HA epitope/His8 cassette (Smk1-HH) was purified using Ni^+2^ beads and analyzed for autophosphorylation (pY209) or recovery of HA (Smk1) by immunoblot analysis as shown in the inset (one sample Wilcoxon *t* test, n = 3, error bars = SD, ∗∗∗; *p* ≤ 0.001). *B*, comparison of sporulation efficiency of strains that were WT at *ISC10* (WT) with *isc10-ΔN110* strains (ΔN110) in backgrounds that contained two copies of *SMK1-HH* (+/+) or one copy of *SMK1-HH* (+/Δ) (Welch’s *t* test, n = 3, error bars = SD, n.s.; no significant difference, ∗∗; *p* ≤ 0.01, ∗∗∗; *p* < 0.001, ∗∗∗∗; *p* ≤ 0.0001). *C*, comparison of Isc10-Myc (WT) and Isc10-ΔN110-Myc (Δ110) levels in cells undergoing meiosis. Extracts prepared from cells collected at the indicated times after being transferred to sporulation medium were analyzed using a Myc antibody and an antibody that recognizes cyclin-dependent kinases (PSTAIRE antibody) as a loading control. The lower member of the doublet is Cdc28 and the upper member of the doublet indicated by the asterisk is Pho85. *D*, quantitation of the data shown in panel (*C*). DAPI, 4′,6-diamidino-2-phenylindole.

We next deleted the first 110 codons from the *ISC10* gene in yeast. Smk1 autophosphorylation was decreased by more than 80% in post-meiotic *isc10-Δ110* cells compared to wild-type (WT) cells (Fig.2A). These results indicate that the YAI is sufficient to inhibit Smk1 autophosphorylation not only in the reconstituted bacterial system but also in yeast cells that are undergoing meiotic development. In addition, these results show that YAI activity in the *isc10-Δ110* mutant is persistent, even past meiosis II when full-length Isc10 is degraded in WT cells (16). Consistent with the persistent inhibition of Smk1 autophosphorylation, the *isc10-Δ110* mutant formed 50% fewer spores than an *ISC10* control strain when two copies of an epitope-tagged form of *SMK1* (*SMK1-HH*) were present and 80% fewer spores when one copy of *SMK1-HH* was present (Fig. 2*B*). These findings genetically connect the meiotic function of *isc10-Δ110* to *SMK1*. The APC/C^Ama1^ E3 ubiquitin ligase targets full-length Isc10 for destruction as cells exit MII. One explanation for the persistent YAI activity in the *isc10-Δ110* strain is that the 1 to 110 interval of Isc10 is required for its regulated degradation. To address this possibility, the WT and Δ110 forms of Isc10 were compared at different times during meiotic development (Fig. 2, *C* and *D*). Both the full-length and deleted forms of Isc10 start to accumulate at 5 h postinduction, when cells are entering MI. As previously reported, the level of full-length Isc10 declines starting around 8 h postinduction, as cells are completing MII, and is undetectable at 24 h. In contrast, the Isc10-Δ110 protein persists after MII has been completed, and it accumulates to higher levels. It has previously been reported that the levels of full-length Isc10 are similarly increased in postmeiotic cells lacking *AMA1* or the core APC/C subunit gene, *SWM1* (16). Taken together, these results suggest that the amino-terminal half of Isc10 is required for the APC/C^Ama1^-dependent destruction of Isc10 as cells complete MII.

### Full-length Isc10 prevents Smk1/Ssp2 from phosphorylating *trans*-substrates while the YAI motif does not

**Figure 3:**
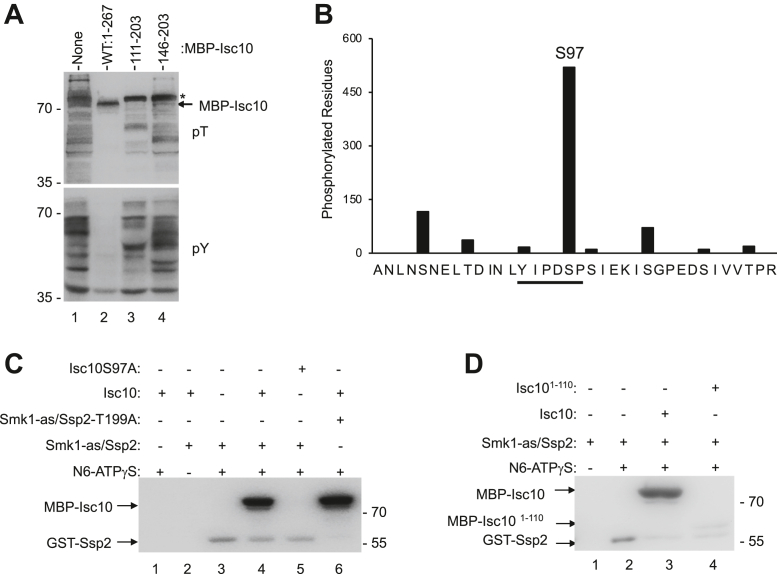
**Smk1 phosphorylates Isc10 on S97.***A*, extracts from bacterial cells expressing Smk1, Ssp2^KAD^-GST, Cak1, and the indicated segments of Isc10 fused to MBP were analyzed by immunoblotting with pT and pY antibodies. The position of MBP-Isc10 is indicated by the arrow. The asterisk indicates a bacterial substrate of Smk1 that migrates slightly slower than MBP-Isc10. This protein is not phosphorylated when full-length MBP-Isc10 is expressed (lane 2) but it is phosphorylated in the absence of MBP-Isc10 (lane 1) or when the YAI is expressed (lanes 3 and 4). These are the same samples that were analyzed in the rightward panel of Fig. 1*A*. *B*, MS/MS analysis of the tryptic peptide of Isc10 containing S97 from bacterial cells producing Smk1, Ssp2^KAD^-GST, Cak1, and MBP-Isc10. Of the 565 tryptic peptides detected, 520 were phosphorylated on S97 (either alone or in combination with other phosphorylated residues). The MS/MS analysis of the entire MBP-Isc10 protein is shown in supporting information (Fig. S1). *C*, Smk1-as/Ssp2 thiophosphorylation assays. Equal amounts of MBP-Isc10 or MBP-Isc10S97A were assayed for thiophosphorylation by Smk1/Ssp2^KAD^-GST (lanes 1–5) or by Smk1/Ssp2^KAD^-T199A (lane 6) complexes purified from bacterial cells that were also coexpressing Cak1. The MBP-Isc10 and Ssp2^KAD^-GST proteins are indicated with arrows. *D*, equal amounts of MBP-Isc10 or MBP-Isc10^1-110^ were assayed for thiophosphorylation by Smk1-as/Ssp2^KAD^-GST. MS, mass spectrometry.

We next examined the effect of full-length Isc10 on Smk1 catalytic output in the bacterial system using phosphospecific antibodies. These experiments revealed that full-length Isc10 prevents Smk1 from phosphorylating numerous proteins on Y and T residues (compare lanes 1 and 2 in Fig. 3*A*). There is an exception however—a protein that comigrates with MBP-Isc10 (indicated by the arrow) that is immunoreactive with the pT antibody. We also analyzed these samples by tandem mass spectrometry (MS). Full-length Isc10 decreased the phosphorylation of bacterial proteins by 75%. Isc10 was phosphorylated by Smk1 on numerous S and T residues with S97 being the major site of phosphorylation (Fig. 3*B* and Supporting Fig. S1). These results show that although Isc10 prevents Smk1/Ssp2 from phosphorylating bacterial proteins, it does not prevent Smk1/Ssp2 from phosphorylating itself. The mAb used in these experiments binds pT residues with high affinity, but it has also been reported to bind pS residues in some peptides with low affinity. Whether the immunoreactivity of Isc10 shown in the upper panel of Fig. 3*A* is a consequence of T residues throughout the protein that the MS/MS analysis indicates are phosphorylated at a low level, crossreactivity with pS97, or a combination of both the pT residues and pS97 has not been determined.

We next tested the phosphorylation of bacterial proteins by Smk1/Ssp2 in cells that coexpressed the Isc10^111-203^ or the Isc10^146-203^ proteins. Although these “minimal YAI” proteins are effective inhibitors of Smk1 autophosphorylation (Fig. 1*A*), bacterial substrate proteins are still phosphorylated on T and Y residues (Fig. 3*A*, lanes 3 and 4). The low Smk1 activity toward *trans*-substrates when full-length Isc10 is expressed and high Smk1 activity toward *trans*-substrates when only the YAI is expressed, suggest that a separate motif in Isc10 plays a role in inhibiting Smk1. The finding that the YAI inhibits autophosphorylation of Y209, but not the phosphorylation of Isc10 or bacterial substrates, suggests that the YAI does not prevent Smk1 from adopting an active kinase conformation. Instead, the YAI specifically inhibits the autophosphorylation of Smk1 (see Discussion).

### Smk1/Ssp2 phosphorylates the Isc10 inhibitor on S97 and the Ssp2 activator on T199

To study the phosphorylation of Isc10 by Smk1 in a more defined system, we used a mutant form of Smk1 in which the “gatekeeper” residue (Q121), that is predicted to limit access to the ATP-binding pocket of the enzyme, was changed to alanine (referred to as *smk1-as1* hereafter). In control experiments, the *smk1-as1* yeast strain formed spores that appeared indistinguishable from those formed by the WT strain. In the presence of bulky purine (ATP) analogs, the *smk1-as1* strain showed a *smk1Δ*-like phenotype (see Experimental procedures). We coexpressed Smk1-as1 with Ssp2^KAD^-GST and Cak1 in bacteria, purified the Smk1-as1/Ssp2^KAD^-GST complex by GSH affinity chromatography, and incubated the purified Smk1-as1/Ssp2^KAD^-GST with and without MBP-Isc10 in a kinase assay buffer containing the as-specific ATP analog N6-benzyl-ATPγS (6-Bn-ATPγS) (24). Thiophosphates transferred by Smk1-as1 to substrate were next alkylated with p-nitrobenzyl mesylate, and the reactions were analyzed by immunoblot analyses using an antibody that recognizes alkylated thiophosphate. These experiments demonstrate that Smk1-as1/Ssp2 can thiophosphorylate MBP-Isc10 *in vitro* (compare lanes 3 and 4 in Fig. 3*C*). We previously showed that Smk1/Ssp2 specifically phosphorylates S or T residues in an extended phosphoconsensus motif, Y-X-P-X-S/T-P (14). S97 in Isc10 is contained in a motif that conforms to this phosphoconsensus sequence (Y-I-P-D-S97-P). Moreover, the MS/MS analysis of Isc10 coexpressed with Smk1, Ssp2, and Cak1 in bacteria showed that the major site of phosphorylation is S97 (Fig. 3*B* and supporting information). Mutation of S97 to alanine eliminated Isc10 thiophosphorylation (compare lanes 4 and 5 in Fig. 3*C*). These data demonstrate that Smk1-as1/Ssp2^KAD^-GST phosphorylates Isc10 exclusively on S97 in the assay conditions tested. While these data indicate that Smk1/Ssp2 can specifically phosphorylate Isc10 on S97, these experiments do not address whether the thiophosphorylation that was observed in these experiments occurred when Isc10 was part of a ternary complex or when Isc10 was unbound (see later).

During these studies, we noticed that Ssp2-GST was also thiophosphorylated. Ssp2 has a motif that conforms to the Smk1 phosphoconsensus except at the -2 position (Y-R-Y-D-T^199^-P). To test whether T199 in Ssp2 is phosphorylated, Smk1-as1/Ssp2^KAD^-T199A-GST was purified from bacteria that also expressed Cak1, then analyzed with thiophosphorylation assays. The T199A substitution eliminated detectable thiophosphorylation of Ssp2 without affecting the phosphorylation of MBP-Isc10 (compare lanes 4 and 6 in Fig. 3*C*). Ssp2^KAD^-GST is present at higher concentrations than Smk1 in the enzymatic preparations used in these experiments. Therefore, similar to the situation with Isc10, we do not know if the Ssp2 that is phosphorylated is free or stably complexed with Smk1 in the holoenzyme. We also do not know whether Ssp2^KAD^-GST that has been phosphorylated in bacteria (and is therefore resistant to the addition of thiophosphate in the *in vitro* reactions) is enriched in the Smk1/Ssp2 complex. Nevertheless, these experiments demonstrate that phosphorylated T199 is not required for Smk1 activity since Smk1/Ssp2-T199A thiophosphorylates MBP-Isc10 similar to Smk1/Ssp2.

We next tested whether deletion of the YAI motif influenced the ability of Smk1-as1/Ssp2 to thiophosphorylate Isc10 on S97 using the phosphorylation of Ssp2 on T199 as a control. For these experiments, identical concentrations of MBP fused to residues 1 to 110 or full-length Isc10 were incubated with Smk1-as1/Ssp2^KAD^-GST and 6-Bn-ATPγS. The products were then alkylated and measured as aforementioned. Although the Isc10 construct lacking the YAI is thiophosphorylated, the reaction takes place at a substantially reduced rate compared to the full-length Isc10 construct (compare lanes 3 and 4 in Fig. 3*D*). These findings indicate that the YAI increases the rate of S97 phosphorylation, likely by tethering Isc10 to the Smk1/Ssp2 complex *via* BD1 and/or 2 and thereby increasing the local concentration of the S97 phosphoconsensus motif. In summary, the data presented in this section demonstrate that Smk1 not only phosphorylates itself (on Y209) but also its activator, Ssp2 (on T199), and its inhibitor, Isc10 (on S97).

### The S97 phosphoconsensus motif prevents Smk1 from phosphorylating Isc10 on other residues

**Figure 4:**
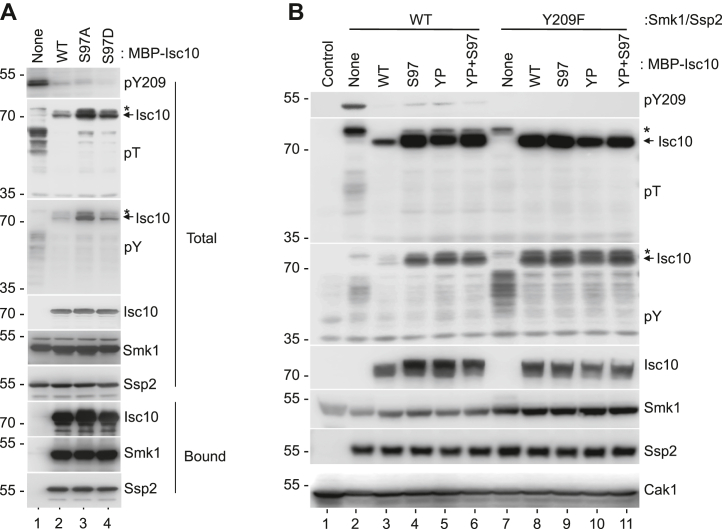
**The S97 phosphoconsensus motif in Isc10 inhibits Smk1 but not Smk1-Y209F.***A*, extracts from bacterial cells expressing Smk1/Ssp2^KAD^-GST, Cak1, and the WT, S97A, and S97D forms of MBP-tagged Isc10 as indicated were analyzed by immunoblotting using pY209, pT, and pY antibodies. Fractions that bound to amylose beads were analyzed to ascertain whether the indicated mutations influenced the ability of Isc10 to form complexes with Smk1 and Ssp2 (Bound). Immunoblots were probed with MBP (Isc10), GST (Ssp2), and Smk1 antibodies. *B*, extracts from bacterial cells expressing Smk1/Ssp2^KAD^-GST and Cak1 (left half - WT) or Smk1-Y209F/Ssp2^KAD^-GST and Cak1 (right half - Y209F) were coexpressed in the absence of MBP-Isc10 (None) or with MBP fused to WT Isc10 (WT); Isc10-S97A (S97); Isc10-Y93A, P95A (YP); or Isc10-S97A,Y93A, P95A (YP + S97). Total extracts were analyzed by immunoblotting using the antibodies indicated on the right side of the blot (described in panel *A*). The asterisk indicates the unidentified bacterial substrate of Smk1 that migrates slightly slower than MBP-Isc10.

To gain insight into the role of S97 phosphorylation in regulating Smk1, MBP-Isc10 carrying nonphosphorylatable (A) or phosphomimetic (D) substitutions of S97 were produced with Smk1, Ssp2^KAD^-GST, and Cak1 in bacteria. The S97A or D substitutions did not influence the ability of Isc10 to inhibit Smk1 autophosphorylation or the ability of Isc10 to form stable complexes with Smk1/Ssp2 (Fig. 4*A*). These data are consistent with the ability of Isc10 deletion mutants lacking S97 (such as Isc10-Δ110) to bind Smk1/Ssp2 and inhibit autophosphorylation (Fig. 1*A*). Since the S97A substitution eliminated detectable phosphorylation of Isc10 by Ssp2/Smk1 *in vitro* (Fig. 3*C*), we expected the S97A substitution to ablate the phosphorylation of Isc10 in the bacterial reconstitution system. However, S97A increased the pT immunoreactivity of Isc10 (compare lanes 2 and 3). This finding suggests that when Isc10 is bound to monophosphorylated Smk1/Ssp2, that the S97A substitution increases the phosphorylation of Isc10 on nonconsensus residues. Consistent with this line of reasoning, the S97A form of Isc10 was also more immunoreactive with a pY antibody than WT Isc10. In addition, the S97A substitution increased the pT and pY immunoreactivity of bacterial proteins. The S97D phosphomimetic mutant also increased the pT and pY immunoreactivity of MBP-Isc10 and bacterial proteins, but these increases were modest compared to those seen in the S97A mutant (compare lanes 3 and 4). Taken together, these findings suggest that phosphorylation of Isc10 on S97 prevents monophosphorylated Smk1 bound to Ssp2 and Isc10 (P-complexes) from phosphorylating other residues in the Isc10 protein and bacterial proteins in *trans*.

To further investigate the role of pS97 in regulating Smk1, the -4Y and -2P residues in the Y-X-P-X-S/T-P phosphoconsensus were changed to A (referred to as the YP mutant later), and the YP mutations were combined with the S97A mutation. The WT or mutant forms of MBP-Isc10 were coexpressed with WT Smk1, Ssp2^KAD^-GST, and Cak1, and phosphorylation was assayed using phosphospecific antibodies (left half of Fig. 4*B* [WT]). As expected, the S97A, YP, and YP+S97A substitutions did not substantially affect the ability of Isc10 to inhibit Smk1 autophosphorylation (pY209 was barely detectable—upper panel of Fig. 4*B*). Like the S97A mutant, the YP substitutions increased pT and pY immunoreactivity of MBP-Isc10 (compare lanes 3–6). The YP + S97A double mutant was indistinguishable from the YP and the S97A single mutants. Furthermore, the YP and S97A mutations reduced the electrophoretic migration of MBP-Isc10 (compare the Isc10 signal in lane 3 with the signals in lanes 4–6). These findings suggest that when the S97-phosphoconsensus motif has been mutated, Smk1 in P-complexes hyperphosphorylates Isc10 on nonconsensus residues. In addition to the effects on Isc10 phosphorylation, the pS97 motif mutations also increased the phosphorylation of bacterial proteins. This is most easily observed by focusing on the pT and pY immunoreactivity of the bacterial protein that migrates slightly slower than MBP-Isc10 in Fig. 4*B* (indicated by asterisks—see also Figs. 3*A* and 4A). Taken together, these observations indicate that the pS97 motif in Isc10 dampens the catalytic output of the poised Smk1/Ssp2/Isc10 complex.

### Smk1-Y209F is resistant to the inhibitory effect of the pS97 motif

We next examined the ability of Smk1-Y209F to phosphorylate the set of MBP-Isc10 phosphoconsensus mutants (right side of Fig. 4*B* [Y209F]). Interestingly, although Smk1-Y209F/Ssp2 is only 30% as active as WT Smk1/Ssp2 in transferring phosphate to substrates (14), the Y209F substitution increased the pT and pY immunoreactivity of WT MBP-Isc10 (compare lanes 3 and 8). Thus, the Y209F substitution in Smk1 and the phosphoconsensus substitutions in Isc10 both increase the phosphorylation of Isc10 on nonconsensus (T and Y) residues (compare lane 8 to lanes 3–6). In addition, the levels of pT and pY immunoreactivity in the Smk1-Y209F samples were not increased further when S97 and/or the YP phosphoconsensus residues in Isc10 were mutated (compare lanes 8–11). Although monophosphorylated Smk1-Y209F is less active than the WT enzyme overall, it is less discriminating against Y and therefore has a higher activity for Y-residues (14) (also compare pT and pY immunoreactivity of lanes 2 and 7). While this might partially explain the increased pY immunoreactivity of Isc10 in the Smk1-Y209F samples, it does not explain the increased pT immunoreactivity of Isc10 or why S97 motif mutations do not further increase Isc10 pT or pY immunoreactivity (compare lane 8 with lanes 9–11). One explanation for these data is that the phosphorylated S97 motif stably interacts with the active site of Smk1 and that the Y209 hydroxyl, which is absent in the Y209F mutant, is required for this interaction. If so, modification of the Y209 hydroxyl by autophosphorylation might also render Smk1 resistant to the inhibitory influence of the phosphorylated S97 motif.

### Isc10 inhibits a mammalian CMGC group kinase, ICK

**Figure 5:**
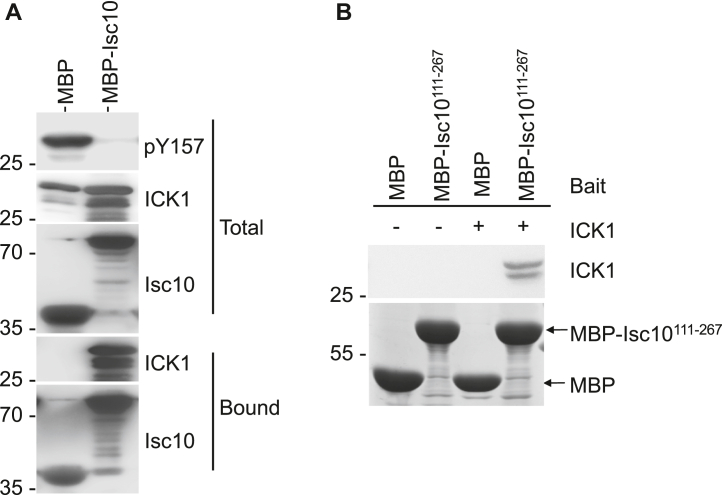
**Isc10 inhibits the autophosphorylation of mammalian ICK (*CILK1*).***A*, extracts from bacterial cells coexpressing the kinase domain of ICK (residues 1–300) with MBP or MBP-Isc10 were analyzed by immunoblotting using an MBP antibody (Isc10), an ICK antiserum, or a pY antibody as indicated. Fractions that bound to amylose beads (Bound) and total extracts (Total) are indicated. *B*, amylose beads bound to MBP or to MBP-Isc10^111-267^ were incubated with extracts from bacteria expressing ICK (+) or not expressing ICK (-). Beads were washed, and the bound fractions were analyzed using an ICK antiserum. MBP and MBP-Isc10^111-267^ levels were visualized by staining with Coomassie blue as indicated.

Intestinal cell kinase (ICK), also referred to as ciliogenesis associated kinase 1 (CILK1), is a member of the ICK/MAK/MOK family of MAPK-related kinases that are phosphorylated by the Cak1 homolog from humans (CCRK) and autophosphorylate their activation loop Y-residues, similar to Smk1 (25, 26). We expressed the kinase domain of ICK in combination with MBP or MBP-Isc10 and assayed the autophosphorylation of its activation loop using a phosphospecific antibody (Fig. 5*A*). These experiments showed that the kinase domain of ICK autophosphorylates its activation loop Y when expressed in bacteria and that MBP-Isc10 inhibits this reaction. We also found that ICK and MBP-Isc10 form a stable complex when coexpressed in bacterial cells (Fig. 5*A*). In addition, purified MBP-Isc10^111-267^ that contains the YAI motif can be used to affinity purify ICK from complex extracts (Fig. 5*B*). Together, these results demonstrate that the YAI motif of Isc10 binds to ICK1 and inhibits the intrinsic ability of ICK1 to autophosphorylate its activation loop.

## Discussion

**Figure 6:**
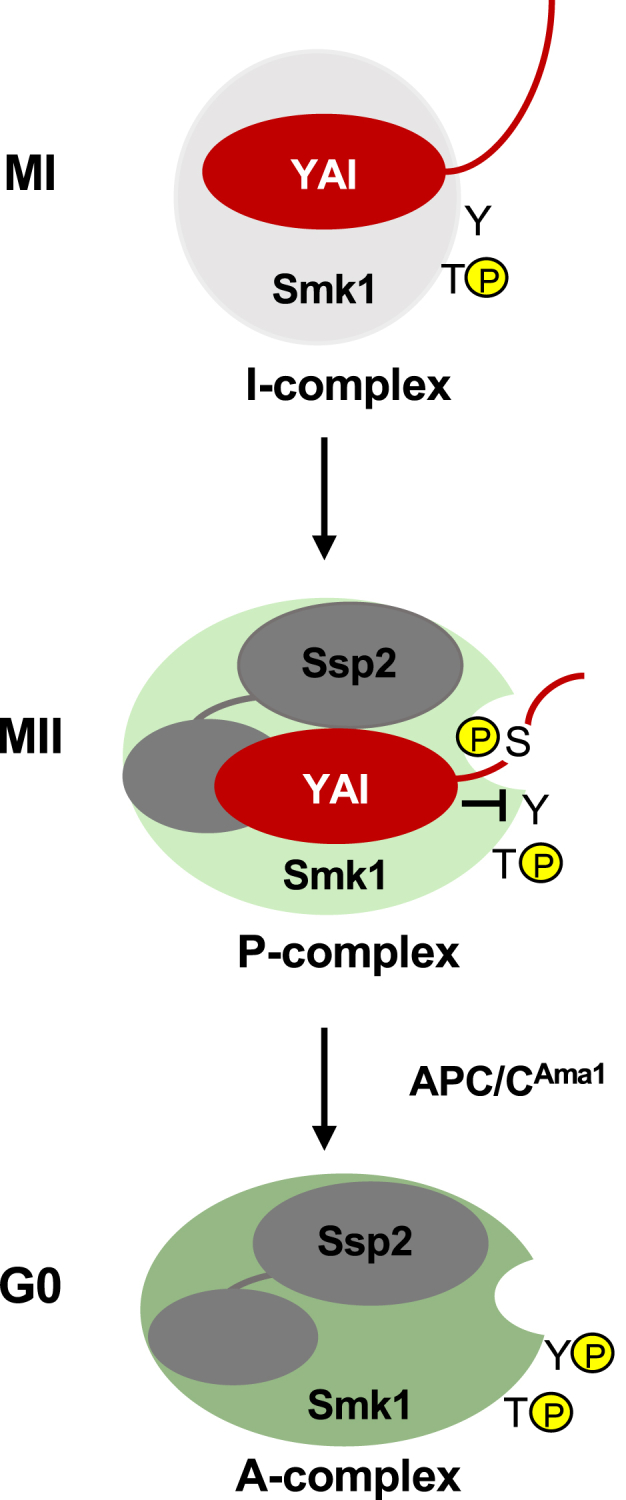
**The role of Isc10 in the inhibited/poised/active (IPA) model for Smk1 activation.** In this model, Isc10 is bound *via* the YAI to an inactive conformation of Smk1 (*light gray* sphere) during MI. The N-terminal half of Isc10 is indicated by the curved *red* line. During MII, Ssp2 binds Smk1/Isc10 through the KARLs (*gray* ellipsoids) and induces an Smk1 conformation that is partially active (*light green*). This change allows Smk1 to phosphorylate the N-terminal half of Isc10 on S97, which prevents Smk1 from phosphorylating other residues in Isc10 and possibly prevents Smk1 from phosphorylating other proteins. At the same time, the YAI specifically prevents Smk1 from autophosphorylating Y209 (inhibitory arm). The YAI also promotes the pS97/active site mechanism by increasing the local concentration of the S97 inhibitory motif. As cells exit MII, Isc10 is ubiquitylated by the APC/C^Ama1^, which leads to the degradation of Isc10, allowing Smk1 to autophosphorylate Y209, thus generating the doubly phosphorylated, fully active conformation of Smk1 (*dark green*).

We previously proposed the IPA model to explain how the activity of Smk1 is coordinated with meiosis (16). In this model, the *SMK1*, *ISC10*, and *SSP2* genes are activated by the middle meiosis-specific transcription factor, Ndt80, as cells enter MI. Smk1 is phosphorylated on its activation loop T by Cak1 during MI and the monophosphorylated MAPK interacts with Isc10 to form heterodimeric I-complexes that disperse throughout the cell (12, 13). Smk1 in I-complexes is catalytically inactive during MI not only because it is bound by the Isc10 inhibitor but because the essential activator, Ssp2, whose mRNA is translationally repressed, is not present (9, 15, 27, 28). Ssp2 mRNA is derepressed during MII, and the Ssp2 protein then binds Smk1/Isc10 to form heterotrimeric P-complexes. As MII is being completed, the APC/C E3 ubiquitin ligase is activated by the meiosis-specific coactivator, Ama1 (29). This leads to the ubiquitylation and subsequent destruction of Isc10, thus generating Ssp2/Smk1 A-complexes that autophosphorylate Smk1’s activation-loop Y. The doubly phosphorylated Smk1 MAPK in A-complexes then triggers key steps in spore formation (10, 11). The IPA pathway thereby couples key steps in differentiation (gametogenesis) to the G0 phase of the cell cycle (Fig. 6). The goal of this study was to elucidate how Isc10 inhibits Smk1.

To understand how Isc10 inhibits Smk1, it is important to consider the role of Ssp2. Ssp2 activates Smk1 *via* two motifs that resemble RNA recognition motifs (RRMs) (30). Both motifs, termed Kinase Activating RRM-Like motifs (KARLs; *gray* ellipsoids in Fig. 6), are required to activate the autophosphorylation of Smk1 on Y209. The KARLs can activate the Smk1-Y209F kinase as well as the WT kinase for substrate phosphorylation, but Smk1-Y209F/Ssp2 is 30% as active as WT Smk1/Ssp2 since autophosphorylation increases catalytic output (14). In the original IPA model, we proposed that Isc10 prevents one of the KARLs in Ssp2 from binding Smk1 (16). According to this model, the APC/C-dependent destruction of Isc10 would allow both KARLs to bind Smk1, thus promoting an active kinase conformation that undergoes autophosphorylation. The discoveries that Isc10 contains a dedicated domain that specifically inhibits autophosphorylation (the YAI) and that Smk1 in P-complexes phosphorylates Isc10, were therefore surprising. These new findings have led to the revised IPA model shown in Fig. 6.

### Relevance to activation loop autophosphorylation of CMGC group kinases

Multiple CMGC kinases have been shown to undergo autophosphorylation of activation loop Ys (2, 3, 4, 5, 6, 31, 32, 33, 34, 35). In many cases, these reactions are thought to be essential for conversion of the kinase from an inactive to an active conformation. How kinases in the inactive conformation phosphorylate themselves presents a paradox. To explain this paradox, it has been proposed that kinases can exist in a unique transitional conformation (the prone to autophosphorylate conformation) (1). We previously showed that Ssp2 partially activates Smk1 even when the activation loop residues are mutated (14). These findings show that a binding protein can induce an active conformation in the absence of activation loop autophosphorylation. The unphosphorylated Fus3 MAPK from yeast can also be triggered to undergo autophosphorylation of its activation loop Y—in this case by a fragment of the Ste5 scaffold protein (36). In addition, DYRK and GSK3ß from humans autophosphorylate their activation loop Y residues as ribosome associated intermediates (4, 5, 6). These reactions require the Hsp90/Cdc37 chaperone complex. The p38α MAPK autophosphorylates its activation loop, and this reaction can be activated by a peptide fragment of TGFß-activated kinase 1 binding protein 1 (TAB1) in response to myocardial ischemia and other conditions (33, 34, 37, 38). The ability of RRM-like motifs, peptide fragments of scaffolds and kinases, as well as chaperones, which do not share obvious sequence/structural similarities to trigger autophosphorylation of inactive kinases, suggest that multiple solutions to the autophosphorylation paradox arose during evolution. These activating mechanisms could involve increasing the affinity of the MAPK for ATP, the orientation of bound ATP, or even the flexibility/structure of the activation loop.

How might Isc10 inhibit the Ssp2-dependent autophosphorylation of Smk1? Two models present themselves. In one model, the YAI prevents autophosphorylation by directly binding to Smk1’s activation loop. In a second model, the YAI acts allosterically. Lee *et al*. have shown that hydroxylation of a proline near the CMGC/MAPK insert in the DYRK1 and GSK3ß kinases can allosterically activate the autophosphorylation of activation loop Ys (6). It is possible that the YAI antagonizes the autophosphorylation of Smk1 by interacting with the CMGC/MAPK insert. Further studies to identify the motifs in Smk1 and Ssp2 that interact with the YAI should shed light on how Isc10 inhibits the autophosphorylation of activation loops in CMGC group kinases.

### Relevance to monophosphorylation and dual-phosphorylation of MAPKs

The well-studied Erk1 and 2 MAPKs from mammals are so much more active *in vitro* when doubly phosphorylated than when singly phosphorylated that they can be considered switches that are in the “on” state only when the enzyme is doubly phosphorylated (39, 40). While there is little doubt that dual phosphorylation triggers high level Erk1/2 activity, there are multiple examples of MAPKs and MAPK-like enzymes that do not conform to this switch-like paradigm. A prominent example is Fus3 from yeast, which exists as singly phosphorylated (on either the T or the Y), as well as doubly phosphorylated isoforms, following stimulation of cells with mating pheromone (41). An interesting feature of this pathway is that although Fus3 is activated by a MAPKK (Ste7) that phosphorylates the activation loop T and Y residues in a canonical MAPK signaling pathway, the Fus3 MAPK can also be triggered to autophosphorylate its activation loop Y by the Ste5 scaffold protein (36). Although the monophosphorylated Fus3 is catalytically active *in vitro* (25%–30% as active as doubly phosphorylated Fus3), it downregulates signaling in a dominant manner *in vivo*. The data presented here suggest that a monophosphorylated MAPK can phosphorylate inhibitory motifs that in turn extinguish catalytic activity. Related interactions may play a role in downregulating other MAPKs and thereby connect monophosphorylated MAPKs to specific signaling outcomes in the cell.

### Relevance to active site inhibition mechanisms

Isc10 is one of 42 proteins in the yeast proteome that contains a perfect Smk1 phosphoconsensus motif Y-X-P-X-S/T-P. Our mutational data show that S97, as well as the -2P and -4Y phosphoconsensus residues, are important for active site inhibition. We propose that Smk1 bound to Isc10 *via* the YAI increases the local concentration of the motif and that binding of Ssp2 then triggers the phosphorylation of Isc10 on S97 (Fig. 6). We further propose that the pS97 motif inhibits the Smk1 active site by a competitive mechanism for substrates that is likely enhanced by the slow disassociation of the motif, due to the interaction of pS97 with Y209 as well as interactions of the -2P and -4Y phosphoconsensus residues with the substrate-binding motif of the kinase. We note that a related substrate-trapping mechanism has been described for the RII regulatory subunit of protein kinase A, which unlike RI regulatory subunits is phosphorylated in a single turnover event that traps pyrophosphate, magnesium, and the phosphoacceptor in the active site of the kinase (42). This allows other signals (Ca^+2^) to trigger activation of the kinase. Our data do not address the stability of the S97p motif/Smk1 active site complex, yet in the context of the Smk1/Ssp2/Isc10 holoenzyme, where the local concentration of the S97 motif is high, it can inhibit the enzyme.

A caveat of the bacterial coexpression experiments is that Smk1 is produced at supraphysiological concentrations. The readout for *trans*-phosphorylation involves high concentrations of nonphysiological substrates whose availability and local concentrations are difficult to assess. It is therefore unclear whether the pS97-dependent suppression of substrate phosphorylation observed in bacteria is relevant to meiotic yeast cells where the concentration of the enzyme is lower. Nevertheless, the concentration of Isc10 relative to Smk1 in the P-complexes produced in bacteria and meiotic yeast cells are identical, and these caveats do not influence the interpretation of the Isc10 phosphorylation data. Thus, while our findings demonstrate that S97p can suppress the phosphorylation of other, presumably nonspecific residues in Isc10, further studies are required to determine whether pS97 plays a physiological role in suppressing the phosphorylation of other downstream targets in meiotic yeast cells. The N-terminal 110 residues of Isc10 appear to play a regulatory role that controls the stability of the protein (Fig. 2, *C* and *D*). One possibility is that the pS97 motif prevents Smk1 from phosphorylating other residues in the N-terminal segment of Isc10 that would otherwise interfere with its ability to be recognized by the APC/C^Ama1^.

### On a switch-like model for Smk1/Ssp2/Isc10

Our data indicate that while Smk1 in P-complexes is inhibited by the S97p motif, the Smk1-Y209F mutant is resistant to the S97p inhibitory mechanism. This indicates that the same -OH that is autophosphorylated by Smk1 is essential for the S97p motif inhibitory mechanism. We speculate that once Y209 has undergone autophosphorylation, the doubly phosphorylated enzyme becomes resistant to inhibition by the S97p motif. If so, the S97p motif mechanism may reinforce the feed-forward switch-like properties that activate the kinase upon completion of the meiotic divisions.

## Experimental procedures

### Bacterial plasmids and coexpression of proteins

Bacterial experiments were carried out with BL-21 DE3 *Escherichia coli* cells using the plasmids listed in Table 1. The methods for coexpression of Smk1, Smk1-Y209F, Cak1, Ssp2^KAD^-GST, and MBP-Isc10 have previously been described (14, 16). To generate the N- or C-terminal deletions in Isc10, fragments containing the Isc10 segments of interest were generated by PCR and inserted into plasmids pZKB12 and pZKB6 (Table 1) using the NheI site that is in frame with the initiator ATG of Isc10 and the NotI site immediately downstream of the Isc10 stop codon. Inserts of all plasmid constructs used in this study were verified by sequencing. Mutations of the S97, Y93, and P95 residues of Isc10 were generated by site-directed mutagenesis (NEB quick change). To create ICK (*CILK1)* constructs, a fragment encoding the kinase domain (codons 1–300) was generated by PCR and cloned into the pACYC-Duet vector using Nde1/KpnI cloning sites to produce pAR130. MBP alone (from pZK11) or MBP-Isc10 (from pZK6) were cloned into pAR130 using the NcoI/NotI cloning sites to generate pAR142 and pAR134.

**Table 1:** Plasmids Used in this study

Plasmid	Description	Source
pJT72	pET-30b + *SMK1*	(14)
pJT111	pET-Duet-1 + *SSP2ΔN137-*GST	(14)
pJT115	pET-Duet-1 + *SSP2ΔN137-*GST + *SMK1*	(14)
pJT122	pACYC-Duet-1 + *CAK1*	(14)
pJT126	pET-Duet-1 + *SSP2ΔN137-*GST + *smk1-as1*	(14)
pJT128	pET-Duet-1 + *SSP2ΔN137-*GST + *smk1-Y209F*	(14)
pZK6	pACYC-Duet-1 + MBP-Pre*-ISC10*	(16)
pZK11	pACYC-Duet-1 + MBP-Pre	(16)
pZK12	pACYC-Duet-1 + MBP-Pre-*ISC10* + *CAK1*	(16)
pAR29	pACYC-Duet-1 + MBP-Pre-*ISC10-S97A*	This study
pAR30	pACYC-Duet-1 + MBP-Pre-*ISC10-S97A* + *CAK1*	This study
pTS2	pACYC-Duet-1 + MBP-Pre-*ISC10Y93A,P95A* + *CAK1*	This study
pTS3	pACYC-Duet-1 + MBP-Pre-*ISC10S97A,Y93A,P95A* + *CAK1*	This study
pAR36	pACYC-Duet-1 + MBP-Pre-*ISC10*^21-267^ + *CAK1*	This study
pAR37	pACYC-Duet-1 + MBP-Pre-*ISC10*^*56-267*^ + *CAK1*	This study
pAR38	pACYC-Duet-1 + MBP-Pre-*ISC10*^*111-267*^ + *CAK1*	This study
pAR39	pACYC-Duet-1 + MBP-Pre-*ISC10*^*1-246*^ + *CAK1*	This study
pAR40	pACYC-Duet-1 + MBP-Pre-*ISC10*^*1-203*^ + *CAK1*	This study
pAR51	pACYC-Duet-1 + MBP-Pre-*ISC10*^*146-267*^ + *CAK1*	This study
pAR52	pACYC-Duet-1 + MBP-Pre-*ISC10*^*181-267*^+ *CAK1*	This study
pAR53	pACYC-Duet-1 + MBP-Pre-*ISC10*^*214-267*^ + *CAK1*	This study
pAR54	pACYC-Duet-1 + MBP-Pre-*ISC10*^*1-170*^ + *CAK1*	This study
pAR55	pACYC-Duet-1 + MBP-Pre-*ISC10*^*1-140*^ + *CAK1*	This study
pAR56	pACYC-Duet-1 + MBP-Pre-*ISC10*^*1-110*^ + *CAK1*	This study
pAR93	pACYC-Duet-1 + MBP-Pre-*ISC10*^*181-267*^	This study
pAR95	pACYC-Duet-1 + MBP-Pre-*ISC10*^*1-170*^	This study
pAR71	pACYC-Duet-1 + MBP-Pre-*ISC10*^*111-203*^ + *CAK1*	This study
pAR72	pACYC-Duet-1 + MBP-Pre-*ISC10*^*146-203*^ + *CAK1*	This study
pAR97	pACYC-Duet-1 + MBP-Pre-*ISC10*^*1-110*^	This study
pAR130	pACYC-Duet-1 + ICK1	This study
pAR142	pACYC-Duet-1 + MBP-Pre + ICK1	This study

### Yeast strains

All yeast strains used in this study were in the SK1 background (Table 2). Cells were grown in YEPD (1% yeast extract, 2% peptone, 2% glucose) supplemented with adenine to 40 μg/ml or SD (0.67% yeast nitrogen base without amino acids, 2% glucose, and nutrients essential for auxotrophic strains) at 30 °C. Sporulation assays were performed by inoculating vegetative cells from YEPD into YEPA (1% yeast extract, 2% peptone, 2% potassium acetate) supplemented with adenine to 40 μg/ml and growing them to a density of 10^7^ cells/ml. Cells were pelleted by centrifugation, washed, and resuspended in sporulation medium (2% potassium acetate, 10 μg/ml adenine, 5 μg/ml histidine, 30 μg/ml leucine, 7.5 μg/ml lysine, 10 μg/ml tryptophan, 5 μg/ml uracil) at 1.5 × 10^7^ cells/ml and incubated in a roller drum at 30 °C.

**Table 2:** Yeast Strains Used in this study

Strain	Genotype	Source
ARY117	MAT**a**/MAT**α***SMK1-HH::LEU2/SMK1-HH::LEU2*	(16)
EWY245	MAT**a**/MAT**α***SMK1-HH::LEU2/SMK1-HH::LEU2* *ISC10ΔN110::hphMX4*/*ISC10ΔN110::hphMX4*	This study
ARY121	MAT**a**/MAT**α***SMK1-HH::LEU2/SMK1-HH::LEU2* *ISC10-13myc::hphMX4/ISC10-13myc:: hphMX4*	This study
ARY190	*MAT****a****/MAT****α****SMK1-HH::LEU2/SMK1-HH::LEU2* *ΔN110-ISC10-13myc::HygB/ΔN110-ISC10-13myc::hphMX4*	This study
ARY203	MAT**a**/MAT**α***SMK1-HH::LEU2/smk1Δ::URA3*	This study
ARY199	MAT**a**/MAT**α***SMK1-HH::LEU2/smk1Δ::URA3* *ISC10ΔN110::hphMX4*/*ISC10ΔN110::hphMX4*	This study

All strains are *MAT****a****/MATα* diploids in the SK1 genetic background that are homozygous for the following markers: *ura3 leu2::hisG trp1::hisG lys2 ho::LYS2*. The strains are histidine auxotrophs that are *his4* and/or *his3*.

To construct the *isc10-Δ110-13myc* strain, the protein coding segment of *isc10-Δ110* was fused to a protein coding segment of *ISC10* tagged at its carboxy terminus with the MYC epitope followed by the hygromycin B marker and the 3′ UTR region of *ISC10* (16).The *isc10-Δ110-13myc*-HygB-3′UTR fragment was transformed into TPY1011 and TPY1013, which were backcrossed and crossmated to generate ARY190.

### Denaturing Smk1-HH purification

Smk1-HH proteins were purified under denaturing conditions as previously described (43). In brief, 2 × 10^8^ cells were collected by centrifugation and lysed by the addition of NaOH. The proteins were precipitated with trichloroacetic acid (TCA) and resuspended in denaturing buffer (6M guanidine hydrochloride, 100 mM NaHPO_4_, 10 mM Tris-Cl pH 8.0). Subsequently, Smk1-HH was bound to nickel beads, eluted in sample buffer containing 200 mM imidazole, and analyzed by gel electrophoresis.

### Immunoblot analyses

For immunoblot analyses of meiotic yeast, equivalent numbers of sporulating cells at different time points were lysed with NaOH, and proteins were precipitated with TCA and solubilized with 8M urea, as described (44). This protocol maximizes the extraction of membrane-associated proteins from TCA precipitates and has been shown to efficiently extract PSM proteins from sporulation cells. Whole cell extracts were electrophoresed on 8% polyacrylamide gels and analyzed by immunoblot analyses with the antibodies described later. In these experiments, Cdc28, whose levels remain relatively stable throughout meiosis was monitored as a loading control.

For immunoblot analyses of bacterial samples, cells were collected by centrifugation and resuspended to 0.005 A^600^ units/μl in loading buffer containing SDS and subjected to four cycles of 30 s boiling/10 s vortexing. Extracts were analyzed by polyacrylamide gel electrophoresis and Coomassie blue staining to assure that equivalent levels of total protein were present in each case. Subsequently, the extracts were analyzed by immunoblot analyses with the antibodies described later.

After electrophoresis, proteins were transferred to Immobilon-P membranes and probed with antibodies. For detection of proteins expressed in the heterologous bacterial expression system, rabbit Smk1 (diluted 1:2000) and pY209 (1:2000) antisera, mouse GST (1:500, Santa Cruz Biotechnology), MBP (1:10,000, NEB), pT (1:1000, CST), and pY (1:1000, CST) mAbs were used. All of these antibodies have been validated for this application in previous studies (14, 16). For detection of proteins from yeast meiotic samples, mouse HA.11 (1:2,000, BioLegend) was used to detect Smk1-HH as previously described (15). Y209p analyses were performed using Smk1-HH that had been purified using denaturing conditions as described previously (9). Isc10-13myc was analyzed with anti-MYC (1:2,000, BioLegend) as described (16). Mouse PSTAIRE (1:10,000, Millipore Sigma) mAb was used to detect Cdc28 (faster migrating) and Pho85 (slower migrating) proteins as loading controls. Horseradish peroxidase–conjugated antibodies raised against mouse (1:7500, BioLegend) or rabbit (1:4000, BioLegend) IgG were used as secondary antibodies and visualized with chemiluminescence substrates using a 1:4 dilution of 1:1 mixture of luminol and H_2_0_2_ (Thermo Fisher Scientific). Western blot images were captured at various exposures, and images within the linear range were quantified using ImageJ version 1.53i (https://imagej.nih.gov/ij/).

### Amylose purification of MBP-tagged Isc10 proteins

For purification of MBP-Isc10 proteins, equivalent numbers of BL-21 DE3 cells were harvested and sonicated four times for 15 s each time in lysis buffer (25 mM Tris-Cl, pH 7.4, 5 mM MgCl_2_, 300 mM NaCl, 1 mM DTT, 0.5% NP-40, and 1 mM PMSF]. Extracts were clarified by centrifugation at 15,000×*g* for 10 min at 4 °C. Soluble fractions were incubated at 4 °C with 80 μl of pre-equilibrated amylose beads (NEB). For immunoblot analyses, the beads were washed with 10 volumes of lysis buffer and eluted in 1× SDS buffer containing 10 mM maltose. For preparing the MBP-Isc10 proteins that were used in kinase assays, beads were washed with 10 volumes of lysis buffer, 10 volumes of kinase buffer (20 mM Hepes, pH 7.4, 100 mM KCl, 10 mM MgCl_2_), eluted in kinase buffer supplemented with 10 mM maltose, and stored in kinase buffer containing 15% (v/v) glycerol at -80 °C.

### Active kinase purification

Ssp2^KAD^-GST/Smk1-as1 was prepared as previously described (14). In brief, 1 l cultures of BL-21 DE3 cells expressing Ssp2^KAD^-GST and Smk1-as1 from the dual IPTG-inducible promoters in pJT126 were harvested and sonicated in lysis buffer (25 mM Tris-Cl, pH 7.4, 5 mM MgCl_2_, 300 mM NaCl, 1 mM DTT, 0.5% NP-40 and 1 mM PMSF) with six rounds of 15 s pulses. The extracts were clarified by centrifugation at 30,000×*g* for 30 min at 4 °C. Soluble fractions were incubated overnight at 4 °C with 500 μl glutathione agarose beads (GenScript) that had been preequilibrated in lysis buffer. The beads were washed with 20 volumes of lysis buffer and 10 volumes of kinase buffer (20 mM HEPES, pH 7.4, 100 mM KCl, 10 mM MgCl_2_) and eluted in kinase buffer supplemented with 20 mM reduced glutathione (Acros Organics), pH 8.0. Protein concentration was quantitated by electrophoresis and Coomassie blue staining using bovine serum albumin (BSA) as a standard. The kinase complexes were frozen in kinase buffer containing 15% v/v glycerol at -80^o^C.

### *In vitro* kinase assays

To generate the analog-sensitive form of Smk1, glutamine (Q) 121 was changed to alanine (A) (*smk1-as1*). *smk1-as1* yeast formed zymolyase-resistant spores that appeared indistinguishable from WT spores in sporulation medium lacked purine analogs. In contrast, the sporulation phenotype of the *smk1-as1* strain was indistinguishable from the *smk1-*Δ sporulation phenotype based on microscopic appearance and zymolyase sensitivity when sporulation media contained 20 μM 1NM-PP1 or 10 μM PPI. The ability of WT cells to form spores was unaffected by both analogs at all concentrations tested. The detailed phenotypic analyses of the *smk1-as1* mutant will be presented elsewhere. To produce the analog-sensitive form of active Smk1 for enzymatic assays, the Q121A mutation was introduced into Smk1 in the ampicillin-selectable Smk1/Ssp2^KAD^-GST coexpression plasmid pJT115 to generate pJT126. pJT126 was cotransformed into BL21-DE3 cells with the chloramphenicol-selectable Cak1 expression plasmid pJT122. Cultures at 18 °C were induced by the addition of 100 μM IPTG, and the Cak1, Smk1-as1, and Ssp2^KAD^-GST coexpressing cells were harvested 12 to 18 h later and Smk1-as1/Ssp2^KAD^-GST was purified using GSH-affinity beads as described previously (14). Thiophosphorylation of substrates was carried out as described (45). In brief, the dually phosphorylated Smk1-as1/Ssp2^KAD^-GST complex was mixed on ice with substrates (MBP-Isc10, MBP-Isc10S97A, or MBP-Isc10^1-110^ as indicated). The mixtures were brought to room temperature (RT) for 5 min and reactions were initiated by the addition of 6-Bn-ATPγS and ATP (to a final concentration of 10 mM and 0.1 mM respectively). Reactions were incubated for 30 min at RT and terminated by the addition of EDTA to a final concentration of 20 mM. Thiophosphates were next alkylated with 2.5 mM p-nitrobenzyl mesylate for 2 h at RT. Samples were resolved by electrophoresis, transferred to membranes, and probed with a rabbit antithiophosphate ester mAb SD2020 (Novus #NBP2-67738 at a 1:20,000 dilution).

### MS

Approximately 100 A^600^ units of BL-21 DE3 cells expressing Smk1, Ssp2^KAD^-GST, and Cak1 with or without Isc10 were lysed in 700 μl lysis buffer (9M urea, 1 mM EDTA, 100 mM Tris-Cl, pH8.0, supplemented with PhosStop phosphatase inhibitors according to manufacturer’s specifications [Roche]). Cells were sonicated for 20 s for two cycles with incubation on ice between the sonication steps. The concentration of the samples was measured by Bradford assay, and proteins were analyzed by MS at the Wistar Proteomics Facility. Five micrograms of each sample was reduced with DTT, alkylated with iodoacetamide, and digested in-solution with trypsin (1:50 enzyme:protein in 2M urea). Starting materials and digests were run on SDS gels and stained with Coomassie blue to confirm trypsin digestion. Digested peptides were purified using Waters SepPak C18 columns. Digested peptides (1.25 mg) were subjected to two sequential TiO2 (GL Sciences) purifications to enrich for phosphorylated peptides. Enriched peptides were analyzed by LC-MS/MS on a Q Exactive HF mass spectrometer using an extended 2 h LC gradient. MS data were searched with full tryptic specificity against the UniProt *E. coli* (BL21-DE3) database (7/3/2021) plus the sequences of Smk1, Ssp2, Cak1, and Isc10, and a common contaminants database using MaxQuant 1.6.17.0. The false discovery rates for protein, peptide, and sites identifications were set at 1%. Phosphorylation (+79.96633 Da) on S, T, and Y were searched on the datasets. For quantitation of total bacterial protein phosphorylation, the iBAQ intensities of yeast proteins were excluded from the analysis.

### Microscopy

End stage meiotic phenotypes in Fig. 2*B* were quantitated by staining cells at 72 h postinduction with 4′,6-diamidino-2-phenylindole. Cells were photographed under wet mount using a Nikon Optiphot equipped for epifluorescence as previously described (10). Sporulation efficiency was analyzed by scoring spore formation in cells that had completed MII.

### Evolutionary conservation analysis

Isc10 protein sequence was compared to sequences in the NCBI protein database using the blast feature. *S. cerevisiae* proteins were excluded from these analyses to remove bias from the *S. cerevisiae*–rich Isc10 sequence database. The NCBI identifier in the retrieved sequences were replaced by species names and re-sorted with *S. cerevisiae* Isc10 sequence as the query (top) sequence for evolutionary relationship within the *Saccharomycetes* family guided by the phylogeny maps (46). The conservation maps were then generated by using ClustalO (47) and visualized in Jalview2 (48).

### Statistical analysis

The statistical analyses were performed in GraphPad Version 9.4.0 (453) (GraphPad Software Inc). For analysis of pY209 levels relative to Smk1 in Figs. 2*A* and 3 independently derived values from ImageJ were analyzed. The values for the WT were set to 100 for comparison between samples. The one sample Wilcoxon *t* test was performed to measure statistical difference between samples. For analysis of spore formation in Fig. 2*B*, approximately 50 cells were counted for each sample in three biological replicates, and the data were plotted and analyzed for statistical difference by Welch’s *t* test (www.graphpad.com). For the immunoblot analyses in Figs. 1, 2C, 3A, and 4, at least two independently derived transformants were assayed with all the antibodies indicated. All of the experiments in Figs. 2, *A* and *B*, and 5 were repeated at least three times.

## Data availability

All data are contained within the article and supporting information.

## Supporting information

This article contains supporting information.

## Conflict of interest

The authors declare that they have no conflicts of interest with the contents of this article.
