# Supplementary material for: Isc10, an inhibitor of the Smk1 MAPK, prevents activation loop autophosphorylation and substrate phosphorylation through separate mechanisms
Source: J Biol Chem. 2022 Sep 3;298(10):102450. doi: 10.1016/j.jbc.2022.102450 (PMC9558048; doi:10.1016/j.jbc.2022.102450)
Supplement: Fig.S1 [file mmc1.pdf]

Database: UP_UP000002032_EcoliBL21DE3_Cust.fasta
Protein ID: C00003
Monoisotopic Mass: 73,141.98

Source: B (non-Razor peptides included)
Protein Name: __CUST MBP-ISC10p (SK1 strain of yeast)
Amino Acid Coverage: 600/648 = 92%

1 648

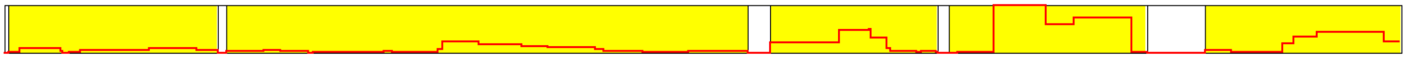

1 MKIEEGKLVI WINGDKGYNG LAEVGKKFEK DTGIKVTVEH PDKLEEKFPQ VAATGDGPDI IFWAHDRFGG YAQSGLLAEI TPDKAFQDKL

91 YPFTWDAVRY NGKLIAYPIA VEALSLIYNK DLLPNPPKTW EEIPALDKEL KAKGKSALMF NLQEPYFTWP LIAADGGYAF KYENGKYDIK

181 DVGVDNAGAK AGLTFLVDLI KNKHMNADTD YSIAEAAFNK GETAMTINGP WAWSNIDTSK VNYGVTVLPT FKGQPSKPFV GVLSAGINAA

271 SPNKELAKEF LENYLLTDEG LEAVNKDKPL GAVALKSYEE ELAKDPRIAA TMENAQKGEI MPNIPQMSAF WYAVRTAVIN AASGRQTVDA

Isc10 start site

361 ALAAAQTNAA ALEVLFQGPA SMDVDERLHQ DENQTHPFSQ KKSSSFLIKE KAATKSKDLE HIRLRDLNFN HRKKLDDKKL AKQIPVKANF

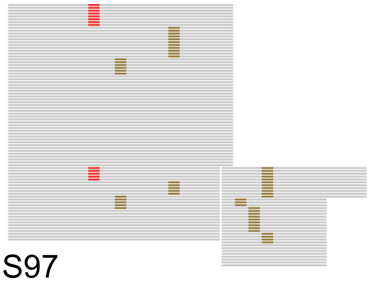

Isc10 S97

451 KKPNEIEARA NLNSNELTDI NLDYIPDSPS IEKISGPEDS IVVTPRNIIH LQSDSDIILE ECEHNYDCSP FYRLFNYENR IEPDDYEAII

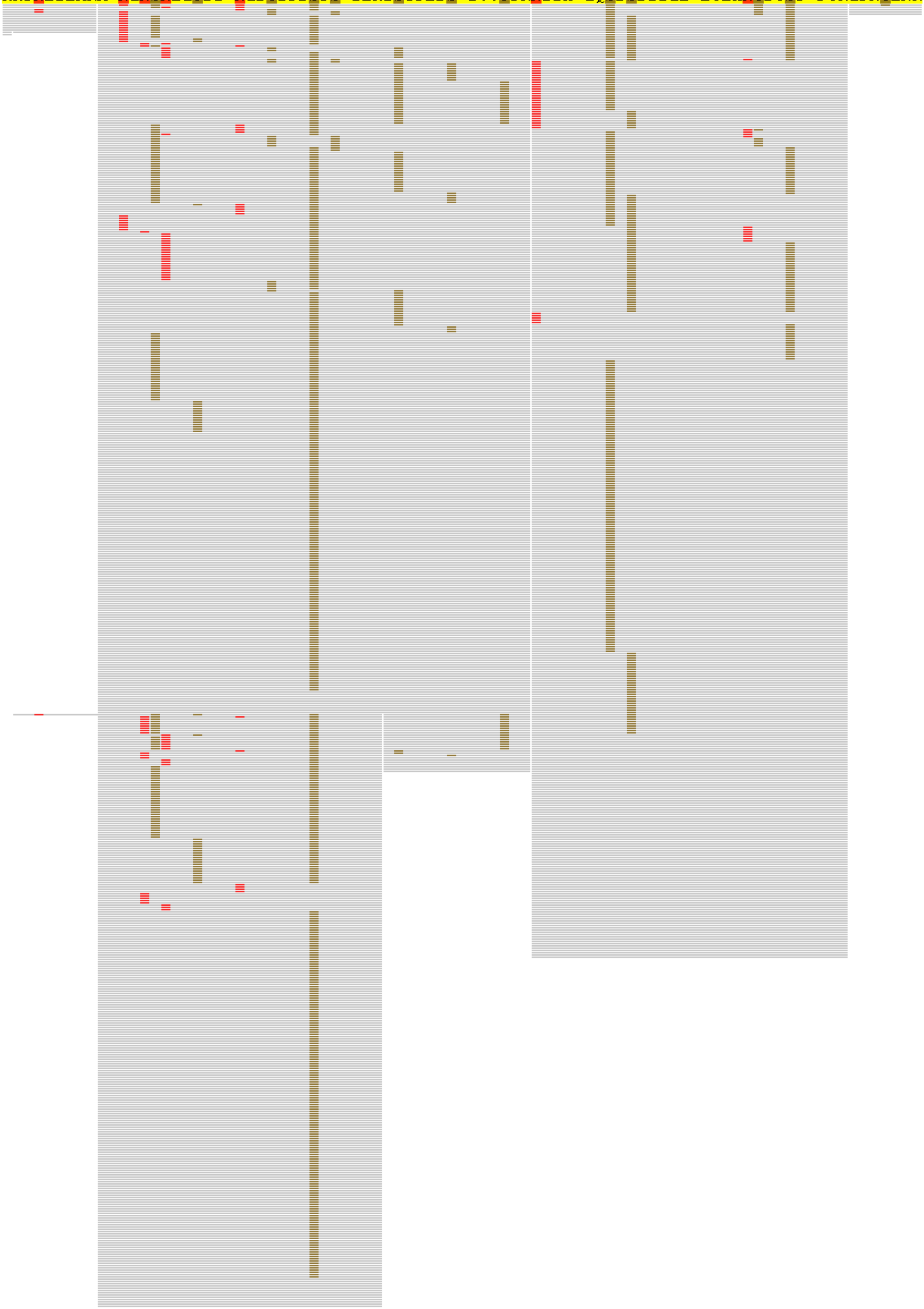

541 NAIITDEIAG TYPVFERELE YQELKSLVRK RDYIMYYFLS RDYRGFFQLK EERTLFYRYP SIAYTSPLRY LDNGSETEQF TGDDDEELQS

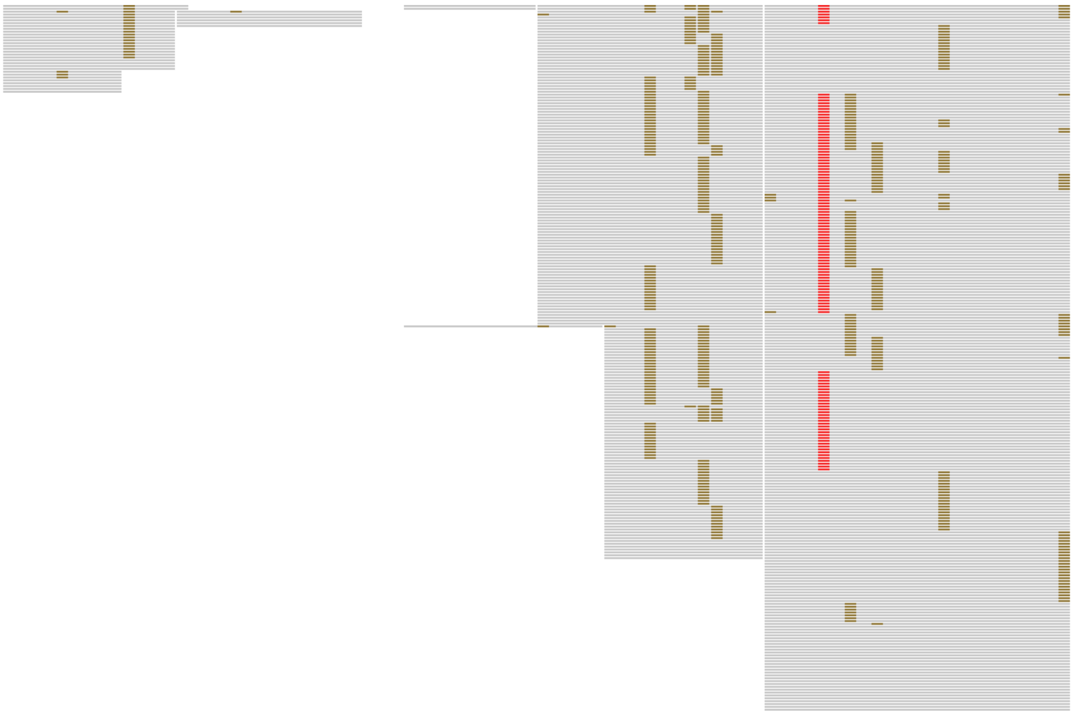

631 FDFENTSSVR TLDSNIWR

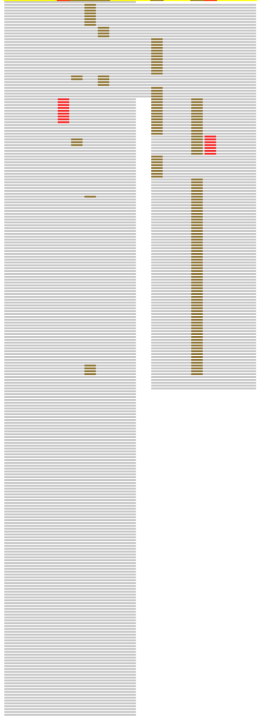

Legends:
+0.98 : Deamidated   +15.99 : Oxidation   +79.97 : Phospho

Peptide:	Count:	Position:	Peptide:	Count:	Position:
AATKSKDLEHIR	2	412-423	LHQDENQTHPFSQKK	6	388-402
AATKSKDLEHIR	6	412-423	LHQDENQTHPFSQKK	32	388-402
AATKSKDLEHIR	6	412-423	LHQDENQTHPFSQKKSSSFLIK	1	388-409
AATKSKDLEHIR	6	412-423	LHQDENQTHPFSQKKSSSFLIK	6	388-409
AFQDKLYPFTWDAVR	5	85-99	LHQDENQTHPFSQKKSSSFLIK	1	388-409
AGLTFLVDLIK	1	191-201	LHQDENQTHPFSQKKSSSFLIK	2	388-409
AGLTFLVDLIK	8	191-201	LHQDENQTHPFSQKKSSSFLIK	8	388-409
AGLTFLVDLIKNK	2	191-203	LHQDENQTHPFSQKKSSSFLIK	6	388-409
AKGKSALMFNLQEPYFTWPLIAADGGYAFK	2	142-171	LHQDENQTHPFSQKKSSSFLIK	4	388-409
ANFKKPNEIEAR	2	448-459	LHQDENQTHPFSQKKSSSFLIK	16	388-409
ANFKKPNEIEAR	2	448-459	LHQDENQTHPFSQKKSSSFLIK	2	388-409
ANFKKPNEIEAR	8	448-459	LHQDENQTHPFSQKKSSSFLIK	1	388-409
ANLNSNELTDINLDYIPDSPSIEK	1	460-483	LHQDENQTHPFSQKKSSSFLIK	16	388-409
ANLNSNELTDINLDYIPDSPSIEK	7	460-483	LHQDENQTHPFSQKKSSSFLIK	3	388-409
ANLNSNELTDINLDYIPDSPSIEK	1	460-483	LHQDENQTHPFSQKKSSSFLIK	4	388-409

ANLNSNELTDINLDYIPDSPSIEK	6	460-483	LIAYPIAVEALSLIYNK	2	104-120
ANLNSNELTDINLDYIPDSPSIEK	1	460-483	LIAYPIAVEALSLIYNK	9	104-120
ANLNSNELTDINLDYIPDSPSIEK	3	460-483	LIAYPIAVEALSLIYNKDLLPNPPK	9	104-128
ANLNSNELTDINLDYIPDSPSIEK	3	460-483	LRDLNFNHR	1	424-432
ANLNSNELTDINLDYIPDSPSIEK	32	460-483	LRDLNFNHR	2	424-432
ANLNSNELTDINLDYIPDSPSIEK	20	460-483	LRDLNFNHRK	4	424-433
ANLNSNELTDINLDYIPDSPSIEK	4	460-483	LRDLNFNHRK	4	424-433
ANLNSNELTDINLDYIPDSPSIEK	5	460-483	LVIWINGDK	2	8-16
ANLNSNELTDINLDYIPDSPSIEK	3	460-483	LVIWINGDK	2	8-16
ANLNSNELTDINLDYIPDSPSIEK	162	460-483	LVIWINGDKGYNGLAEVGK	8	8-26
ANLNSNELTDINLDYIPDSPSIEK	13	460-483	LVIWINGDKGYNGLAEVGK	2	8-26
ANLNSNELTDINLDYIPDSPSIEKISGPEDSIVVTPR	1	460-496	LVIWINGDKGYNGLAEVGK	12	8-26
ANLNSNELTDINLDYIPDSPSIEKISGPEDSIVVTPR	1	460-496	LVIWINGDKGYNGLAEVGK	4	8-26
ANLNSNELTDINLDYIPDSPSIEKISGPEDSIVVTPR	1	460-496	LVIWINGDKGYNGLAEVGKK	2	8-27
ANLNSNELTDINLDYIPDSPSIEKISGPEDSIVVTPR	2	460-496	LVIWINGDKGYNGLAEVGKK	3	8-27
ANLNSNELTDINLDYIPDSPSIEKISGPEDSIVVTPR	10	460-496	LVIWINGDKGYNGLAEVGKK	7	8-27
ANLNSNELTDINLDYIPDSPSIEKISGPEDSIVVTPR	2	460-496	LVIWINGDKGYNGLAEVGKK	4	8-27
ANLNSNELTDINLDYIPDSPSIEKISGPEDSIVVTPR	1	460-496	LYPFTWDAVR	3	90-99
ANLNSNELTDINLDYIPDSPSIEKISGPEDSIVVTPR	1	460-496	NIIHLQSDSDIILEECEHNYDCSPFYR	5	497-523
ANLNSNELTDINLDYIPDSPSIEKISGPEDSIVVTPR	2	460-496	NIIHLQSDSDIILEECEHNYDCSPFYR	19	497-523
ANLNSNELTDINLDYIPDSPSIEKISGPEDSIVVTPR	3	460-496	NIIHLQSDSDIILEECEHNYDCSPFYR	1	497-523
ANLNSNELTDINLDYIPDSPSIEKISGPEDSIVVTPR	2	460-496	NIIHLQSDSDIILEECEHNYDCSPFYR	22	497-523
ANLNSNELTDINLDYIPDSPSIEKISGPEDSIVVTPR	8	460-496	NIIHLQSDSDIILEECEHNYDCSPFYR	8	497-523
ANLNSNELTDINLDYIPDSPSIEKISGPEDSIVVTPR	19	460-496	NIIHLQSDSDIILEECEHNYDCSPFYR	1	497-523
ANLNSNELTDINLDYIPDSPSIEKISGPEDSIVVTPR	4	460-496	NIIHLQSDSDIILEECEHNYDCSPFYR	3	497-523
ANLNSNELTDINLDYIPDSPSIEKISGPEDSIVVTPR	1	460-496	NIIHLQSDSDIILEECEHNYDCSPFYR	4	497-523
ANLNSNELTDINLDYIPDSPSIEKISGPEDSIVVTPR	5	460-496	NIIHLQSDSDIILEECEHNYDCSPFYR	21	497-523
ANLNSNELTDINLDYIPDSPSIEKISGPEDSIVVTPR	2	460-496	NIIHLQSDSDIILEECEHNYDCSPFYR	14	497-523
ANLNSNELTDINLDYIPDSPSIEKISGPEDSIVVTPR	18	460-496	NIIHLQSDSDIILEECEHNYDCSPFYR	7	497-523
ANLNSNELTDINLDYIPDSPSIEKISGPEDSIVVTPR	5	460-496	NIIHLQSDSDIILEECEHNYDCSPFYR	31	497-523
ANLNSNELTDINLDYIPDSPSIEKISGPEDSIVVTPR	1	460-496	NIIHLQSDSDIILEECEHNYDCSPFYR	5	497-523
ANLNSNELTDINLDYIPDSPSIEKISGPEDSIVVTPR	4	460-496	NIIHLQSDSDIILEECEHNYDCSPFYR	16	497-523
ANLNSNELTDINLDYIPDSPSIEKISGPEDSIVVTPR	7	460-496	NIIHLQSDSDIILEECEHNYDCSPFYR	129	497-523
ANLNSNELTDINLDYIPDSPSIEKISGPEDSIVVTPR	1	460-496	NIIHLQSDSDIILEECEHNYDCSPFYR	36	497-523
ANLNSNELTDINLDYIPDSPSIEKISGPEDSIVVTPR	21	460-496	NIIHLQSDSDIILEECEHNYDCSPFYR	99	497-523
ANLNSNELTDINLDYIPDSPSIEKISGPEDSIVVTPR	4	460-496	NKHMNADTDYSIAEAAFNK	6	202-220
ANLNSNELTDINLDYIPDSPSIEKISGPEDSIVVTPR	1	460-496	NKHMNADTDYSIAEAAFNK	2	202-220
ANLNSNELTDINLDYIPDSPSIEKISGPEDSIVVTPR	15	460-496	NKHMNADTDYSIAEAAFNK	4	202-220
ANLNSNELTDINLDYIPDSPSIEKISGPEDSIVVTPR	3	460-496	NKHMNADTDYSIAEAAFNK	2	202-220
ANLNSNELTDINLDYIPDSPSIEKISGPEDSIVVTPR	30	460-496	NKHMNADTDYSIAEAAFNK	7	202-220
ANLNSNELTDINLDYIPDSPSIEKISGPEDSIVVTPR	14	460-496	NKHMNADTDYSIAEAAFNKGETAMTINGPWAWSNIDTSK	2	202-240
ANLNSNELTDINLDYIPDSPSIEKISGPEDSIVVTPR	114	460-496	NKHMNADTDYSIAEAAFNKGETAMTINGPWAWSNIDTSK	1	202-240
ANLNSNELTDINLDYIPDSPSIEKISGPEDSIVVTPR	10	460-496	NKHMNADTDYSIAEAAFNKGETAMTINGPWAWSNIDTSK	1	202-240
DKPLGAVALK	4	297-306	NKHMNADTDYSIAEAAFNKGETAMTINGPWAWSNIDTSK	1	202-240
DLLPNPPK	7	121-128	NKHMNADTDYSIAEAAFNKGETAMTINGPWAWSNIDTSK	1	202-240
DLLPNPPKTWEEIPALDK	2	121-138	NKHMNADTDYSIAEAAFNKGETAMTINGPWAWSNIDTSK	3	202-240
DLLPNPPKTWEEIPALDKELK	3	121-141	NKHMNADTDYSIAEAAFNKGETAMTINGPWAWSNIDTSK	4	202-240
DLLPNPPKTWEEIPALDKELK	6	121-141	NKHMNADTDYSIAEAAFNKGETAMTINGPWAWSNIDTSK	10	202-240
DLNFNHR	1	426-432	QIPVKANFK	2	443-451
DLNFNHR	2	426-432	QTVDAALAAAQTNAAALEVLFQGPASMDVDER	7	356-387
DLNFNHRK	1	426-433	QTVDAALAAAQTNAAALEVLFQGPASMDVDER	1	356-387
DTGIKVTVEHPDK	1	31-43	QTVDAALAAAQTNAAALEVLFQGPASMDVDER	7	356-387
DTGIKVTVEHPDKLEEK	1	31-47	QTVDAALAAAQTNAAALEVLFQGPASMDVDERLHQDENQTHPFSQK	4	356-401
DTGIKVTVEHPDKLEEK	5	31-47	QTVDAALAAAQTNAAALEVLFQGPASMDVDERLHQDENQTHPFSQK	13	356-401
DYRGFFQLKEER	1	582-593	QTVDAALAAAQTNAAALEVLFQGPASMDVDERLHQDENQTHPFSQK	1	356-401
DYRGFFQLKEER	2	582-593	QTVDAALAAAQTNAAALEVLFQGPASMDVDERLHQDENQTHPFSQK	8	356-401
EFLENYLLTDEGLEAVNK	6	279-296	QTVDAALAAAQTNAAALEVLFQGPASMDVDERLHQDENQTHPFSQKK	4	356-402
EFLENYLLTDEGLEAVNKDKPLGAVALK	1	279-306	QTVDAALAAAQTNAAALEVLFQGPASMDVDERLHQDENQTHPFSQKK	1	356-402
EFLENYLLTDEGLEAVNKDKPLGAVALK	1	279-306	QTVDAALAAAQTNAAALEVLFQGPASMDVDERLHQDENQTHPFSQKK	2	356-402
EFLENYLLTDEGLEAVNKDKPLGAVALK	7	279-306	QTVDAALAAAQTNAAALEVLFQGPASMDVDERLHQDENQTHPFSQKK	1	356-402
ELAKEFLENYLLTDEGLEAVNK	3	275-296	QTVDAALAAAQTNAAALEVLFQGPASMDVDERLHQDENQTHPFSQKK	8	356-402
ELEYQELK	3	558-565	QTVDAALAAAQTNAAALEVLFQGPASMDVDERLHQDENQTHPFSQKK	1	356-402
ELEYQELK	5	558-565	QTVDAALAAAQTNAAALEVLFQGPASMDVDERLHQDENQTHPFSQKK	2	356-402
ELEYQELKSLVR	1	558-569	QTVDAALAAAQTNAAALEVLFQGPASMDVDERLHQDENQTHPFSQKK	34	356-402
ELEYQELKSLVR	16	558-569	QTVDAALAAAQTNAAALEVLFQGPASMDVDERLHQDENQTHPFSQKK	2	356-402
ELEYQELKSLVR	4	558-569	QTVDAALAAAQTNAAALEVLFQGPASMDVDERLHQDENQTHPFSQKK	6	356-402
ELEYQELKSLVRK	5	558-570	QTVDAALAAAQTNAAALEVLFQGPASMDVDERLHQDENQTHPFSQKK	20	356-402
FGGYAQSGLLAEITPDK	2	68-84	SALMFNLQEPYFTWPLIAADGGYAFKYENGKYDIK	2	146-180
FGGYAQSGLLAEITPDK	5	68-84	SALMFNLQEPYFTWPLIAADGGYAFKYENGKYDIK	1	146-180
FGGYAQSGLLAEITPDK	2	68-84	SKDLEHIR	1	416-423
FGGYAQSGLLAEITPDKAFQDK	3	68-89	SKDLEHIR	4	416-423
FGGYAQSGLLAEITPDKAFQDK	1	68-89	SSSFLIK	8	403-409
FGGYAQSGLLAEITPDKAFQDK	8	68-89	SSSFLIK	24	403-409
FGGYAQSGLLAEITPDKAFQDK	5	68-89	SSSFLIK	2	403-409

FGGYAQSGLLAEITPDKAFQDKLYPFFTWDAVR	4	68-99	SSSFLIKEK	1	403-411
FGGYAQSGLLAEITPDKAFQDKLYPFFTWDAVR	5	68-99	SSSFLIKEK	14	403-411
FGGYAQSGLLAEITPDKAFQDKLYPFFTWDAVR	9	68-99	SSSFLIKEK	2	403-411
FGGYAQSGLLAEITPDKAFQDKLYPFFTWDAVR	5	68-99	SSSFLIKEK	1	403-411
FPQVAATGDGPDIIFWAHDR	3	48-67	SSSFLIKEKAATK	5	403-415
FPQVAATGDGPDIIFWAHDR	3	48-67	SYEEELAKDPR	2	307-317
FPQVAATGDGPDIIFWAHDRFGGYAQSGLLAEITPDK	1	48-84	SYEEELAKDPR	11	307-317
FPQVAATGDGPDIIFWAHDRFGGYAQSGLLAEITPDKAFQDK	6	48-89	SYEEELAKDPRIAATMENAQK	1	307-327
GEIMPNIPQMSAFWYAVR	6	328-345	TLDSNIWR	13	641-648
GFFQLKEER	2	585-593	TLDSNIWR	7	641-648
GFFQLKEERTLFYR	1	585-598	TLDSNIWR	8	641-648
GKSALMFNLQEPYFTWPLIAADGGYAFK	1	144-171	TLDSNIWR	69	641-648
GKSALMFNLQEPYFTWPLIAADGGYAFK	1	144-171	TLDSNIWR	5	641-648
GQPSKPFVGVLSAGINAASPNK	1	253-274	TLFYRYPSIAYTSPLR	1	594-609
GQPSKPFVGVLSAGINAASPNK	9	253-274	TLFYRYPSIAYTSPLR	3	594-609
GQPSKPFVGVLSAGINAASPNK	3	253-274	TLFYRYPSIAYTSPLR	1	594-609
GQPSKPFVGVLSAGINAASPNK	9	253-274	TLFYRYPSIAYTSPLR	1	594-609
GQPSKPFVGVLSAGINAASPNK	4	253-274	TLFYRYPSIAYTSPLR	6	594-609
GQPSKPFVGVLSAGINAASPNKELAK	6	253-278	TLFYRYPSIAYTSPLR	4	594-609
GQPSKPFVGVLSAGINAASPNKELAK	3	253-278	TLFYRYPSIAYTSPLR	11	594-609
GQPSKPFVGVLSAGINAASPNKELAK	11	253-278	TLFYRYPSIAYTSPLR	5	594-609
GQPSKPFVGVLSAGINAASPNKELAK	3	253-278	TLFYRYPSIAYTSPLR	19	594-609
GQPSKPFVGVLSAGINAASPNKELAK	9	253-278	TLFYRYPSIAYTSPLR	4	594-609
GQPSKPFVGVLSAGINAASPNKELAK	4	253-278	TLFYRYPSIAYTSPLR	20	594-609
GQPSKPFVGVLSAGINAASPNKELAK	6	253-278	TLFYRYPSIAYTSPLR	18	594-609
GYNGLAEVGK	3	17-26	TLFYRYPSIAYTSPLR	16	594-609
GYNGLAEVGK	2	17-26	TLFYRYPSIAYTSPLR	5	594-609
HMNADTDYSIAEAAFNK	1	204-220	TLFYRYPSIAYTSPLRYLDNGSETEQFTGDDDEELQSFDFENTSSVR	1	594-640
HMNADTDYSIAEAAFNK	3	204-220	TWEEIPALDKELK	11	129-141
HMNADTDYSIAEAAFNK	3	204-220	VNYGVTVLPTFK	4	241-252
HMNADTDYSIAEAAFNKGETAMTINGPWAWSNIDTSK	1	204-240	VNYGVTVLPTFKGQPSKPFVGVLSAGINAASPNK	3	241-274
HMNADTDYSIAEAAFNKGETAMTINGPWAWSNIDTSK	1	204-240	VTVEHPDKLEEK	4	36-47
HMNADTDYSIAEAAFNKGETAMTINGPWAWSNIDTSK	1	204-240	VTVEHPDKLEEKFPQVAATGDGPDIIFWAHDR	11	36-67
HMNADTDYSIAEAAFNKGETAMTINGPWAWSNIDTSK	5	204-240	VTVEHPDKLEEKFPQVAATGDGPDIIFWAHDR	13	36-67
HMNADTDYSIAEAAFNKGETAMTINGPWAWSNIDTSKVNYGVTVLPTFK	1	204-252	YDIKDVGVDNAGAK	1	177-190
HMNADTDYSIAEAAFNKGETAMTINGPWAWSNIDTSKVNYGVTVLPTFK	10	204-252	YDIKDVGVDNAGAK	4	177-190
HMNADTDYSIAEAAFNKGETAMTINGPWAWSNIDTSKVNYGVTVLPTFK	2	204-252	YENGKYDIK	1	172-180
HMNADTDYSIAEAAFNKGETAMTINGPWAWSNIDTSKVNYGVTVLPTFK	13	204-252	YENGKYDIKDVGVDNAGAK	3	172-190
HMNADTDYSIAEAAFNKGETAMTINGPWAWSNIDTSKVNYGVTVLPTFK	4	204-252	YENGKYDIKDVGVDNAGAK	4	172-190
HMNADTDYSIAEAAFNKGETAMTINGPWAWSNIDTSKVNYGVTVLPTFK	3	204-252	YLDNGSETEQFTGDDDEELQSFDFENTSSVR	1	610-640
HMNADTDYSIAEAAFNKGETAMTINGPWAWSNIDTSKVNYGVTVLPTFK	15	204-252	YLDNGSETEQFTGDDDEELQSFDFENTSSVR	8	610-640
HMNADTDYSIAEAAFNKGETAMTINGPWAWSNIDTSKVNYGVTVLPTFK	8	204-252	YLDNGSETEQFTGDDDEELQSFDFENTSSVR	3	610-640
HMNADTDYSIAEAAFNKGETAMTINGPWAWSNIDTSKVNYGVTVLPTFK	18	204-252	YLDNGSETEQFTGDDDEELQSFDFENTSSVR	2	610-640
IAATMENAQKGEIMPNIPQMSAFWYAVR	1	318-345	YLDNGSETEQFTGDDDEELQSFDFENTSSVR	3	610-640
IAATMENAQKGEIMPNIPQMSAFWYAVR	2	318-345	YLDNGSETEQFTGDDDEELQSFDFENTSSVR	3	610-640
IAATMENAQKGEIMPNIPQMSAFWYAVR	3	318-345	YLDNGSETEQFTGDDDEELQSFDFENTSSVR	8	610-640
IAATMENAQKGEIMPNIPQMSAFWYAVR	3	318-345	YLDNGSETEQFTGDDDEELQSFDFENTSSVR	6	610-640
IAATMENAQKGEIMPNIPQMSAFWYAVR	1	318-345	YLDNGSETEQFTGDDDEELQSFDFENTSSVR	1	610-640
IAATMENAQKGEIMPNIPQMSAFWYAVR	8	318-345	YLDNGSETEQFTGDDDEELQSFDFENTSSVR	2	610-640
IEEGKLVIWINGDKGYNGLAEVGK	4	3-26	YLDNGSETEQFTGDDDEELQSFDFENTSSVR	1	610-640
IEEGKLVIWINGDKGYNGLAEVGK	7	3-26	YLDNGSETEQFTGDDDEELQSFDFENTSSVR	3	610-640
IEEGKLVIWINGDKGYNGLAEVGK	1	3-26	YLDNGSETEQFTGDDDEELQSFDFENTSSVR	20	610-640
ISGPEDSIVVTPR	16	484-496	YLDNGSETEQFTGDDDEELQSFDFENTSSVR	15	610-640
ISGPEDSIVVTPR	2	484-496	YLDNGSETEQFTGDDDEELQSFDFENTSSVR	1	610-640
ISGPEDSIVVTPR	1	484-496	YLDNGSETEQFTGDDDEELQSFDFENTSSVR	8	610-640
ISGPEDSIVVTPR	7	484-496	YLDNGSETEQFTGDDDEELQSFDFENTSSVR	7	610-640
KFEKDTGIK	1	27-35	YLDNGSETEQFTGDDDEELQSFDFENTSSVR	1	610-640
KLAKQIPVK	1	439-447	YLDNGSETEQFTGDDDEELQSFDFENTSSVR	4	610-640
KPNEIEAR	1	452-459	YLDNGSETEQFTGDDDEELQSFDFENTSSVR	35	610-640
KPNEIEARANLNSNELTDINLDYIPDSPSIEK	1	452-483	YLDNGSETEQFTGDDDEELQSFDFENTSSVR	21	610-640
KRDYIMYYFLSR	1	570-581	YLDNGSETEQFTGDDDEELQSFDFENTSSVR	25	610-640
KRDYIMYYFLSR	5	570-581	YLDNGSETEQFTGDDDEELQSFDFENTSSVR	7	610-640
KSSSFLIK	3	402-409	YLDNGSETEQFTGDDDEELQSFDFENTSSVR	1	610-640
KSSSFLIK	9	402-409	YLDNGSETEQFTGDDDEELQSFDFENTSSVR	30	610-640
KSSSFLIK	4	402-409	YLDNGSETEQFTGDDDEELQSFDFENTSSVRTLDSNIWR	7	610-648
KSSSFLIK	8	402-409	YLDNGSETEQFTGDDDEELQSFDFENTSSVRTLDSNIWR	1	610-648
KSSSFLIKEK	2	402-411	YLDNGSETEQFTGDDDEELQSFDFENTSSVRTLDSNIWR	1	610-648
KSSSFLIKEK	5	402-411	YLDNGSETEQFTGDDDEELQSFDFENTSSVRTLDSNIWR	3	610-648
KSSSFLIKEK	1	402-411	YLDNGSETEQFTGDDDEELQSFDFENTSSVRTLDSNIWR	13	610-648
KSSSFLIKEK	15	402-411	YLDNGSETEQFTGDDDEELQSFDFENTSSVRTLDSNIWR	2	610-648
KSSSFLIKEK	11	402-411	YLDNGSETEQFTGDDDEELQSFDFENTSSVRTLDSNIWR	2	610-648
KSSSFLIKEK	1	402-411	YLDNGSETEQFTGDDDEELQSFDFENTSSVRTLDSNIWR	4	610-648
LFNYENR	1	524-530	YPSIAYTSPLR	1	599-609
LFNYENR	4	524-530	YPSIAYTSPLR	21	599-609
LHQDENQTHPFSQK	5	388-401	YPSIAYTSPLR	6	599-609

LHQDENQTHPFSQK	5	388-401	YPSIAYTSPLR	1	599-609
LHQDENQTHPFSQK	5	388-401	YPSIAYTSPLR	5	599-609
LHQDENQTHPFSQK	11	388-401	YPSIAYTSPLR	13	599-609
LHQDENQTHPFSQKK	11	388-402	YPSIAYTSPLR	16	599-609
LHQDENQTHPFSQKK	4	388-402	YPSIAYTSPLR	12	599-609
LHQDENQTHPFSQKK	11	388-402	YPSIAYTSPLR	7	599-609
LHQDENQTHPFSQKK	11	388-402			

Figure S1. Mass spectrometry of sites in MBP-Isc10 that are phosphorylated by Smk1 in *E. coli.* Total proteins from cells expressing Smk1, Ssp2137-371-GST, Cak1, and MBP-Isc10 were digested with trypsin and peptides were purified by C18 HPLC chromatography followed by two rounds of TiO2 chromatography. Purified material was analyzed by LC-MS/MS as described in the Experimental Procedures section. Red, deamidation (+0.98); green, oxidation (+15.99); brown, phosphorylation (+79.97). The start site methionine (M) of Isc10 (numbered 382) and the S97 phosphoacceptor (numbered 478) are indicated.
